# Association of genes in hereditary metabolic diseases with diagnosis, prognosis, and treatment outcomes in gastric cancer

**DOI:** 10.3389/fimmu.2023.1289700

**Published:** 2023-11-09

**Authors:** Yiping Li, Xiaoqin Li, Yufei Yang, Xuehan Qiao, Qing Tao, Chen Peng, Miao Han, Kebin Dong, Min Xu, Deqiang Wang, Gaohua Han

**Affiliations:** ^1^ Department of Oncology, The Affiliated Taizhou People’s Hospital of Nanjing Medical University, Taizhou, China; ^2^ Department of Oncology, Digestive Disease Institute & Cancer Institute of Jiangsu University, Affiliated Hospital of Jiangsu University, Zhenjiang, China; ^3^ Department of Gastroenterology, Digestive Disease Institute of Jiangsu University, Affiliated Hospital of Jiangsu University, Zhenjiang, China

**Keywords:** gastric cancer, metabolism, gene, immunotherapy, prognosis

## Abstract

**Background:**

Aberrant metabolism is a major hallmark of cancers and hereditary diseases. Genes associated with inborn metabolic errors may also play roles in cancer development. This study evaluated the overall impact of these genes on gastric cancer (GC).

**Methods:**

In total, 162 genes involved in 203 hereditary metabolic diseases were identified in the Human Phenotype Ontology database. Clinical and multi-omic data were acquired from the GC cohort of the Affiliated Hospital of Jiangsu University and other published cohorts. A 4-gene and 32-gene signature was established for diagnosis and prognosis or therapeutic prediction, respectively, and corresponding abnormal metabolism scores (AMscores) were calculated.

**Results:**

The diagnostic AMscore showed high sensitivity (0.88-1.00) and specificity (0.89-1.00) to distinguish between GC and paired normal tissues, with area under the receiver operating characteristic curve (AUC) ranging from 0.911 to 1.000 in four GC cohorts. The prognostic or predictive AMscore was an independent predictor of overall survival (OS) in five GC cohorts and a predictor of the OS and disease-free survival benefit of postoperative chemotherapy or chemoradiotherapy in one GC cohort with such data. The AMscore adversely impacts immune biomarkers, including tumor mutation burden, tumor neoantigen burden, microsatellite instability, programmed death-ligand 1 protein expression, tumor microenvironment score, T cell receptor clonality, and immune cell infiltration detected by multiplex immunofluorescence staining. The AUC of the AMscore for predicting immunotherapy response ranging from 0.780 to 0.964 in four cohorts involving GC, urothelial cancer, melanoma, and lung cancer. The objective response rates in the low and high AMscore subgroups were 78.6% and 3.2%, 40.4% and 7%, 52.6% and 0%, and 72.7% and 0%, respectively (all p<0.001). In cohorts with survival data, a high AMscore was hazardous for OS or progression-free survival, with hazard ratios ranged from 5.79 to 108.59 (all p<0.001). Importantly, the AMscore significantly improved the prediction of current immune biomarkers for both response and survival, thus redefining the advantaged and disadvantaged immunotherapy populations.

**Conclusions:**

Signatures based on genes associated with hereditary metabolic diseases and their corresponding scores could be used to guide the diagnosis and treatment of GC. Therefore, further validation is required.

## 1 Introduction

Gastric cancer (GC) is one of the most prevalent and fatal cancers worldwide, ranking fifth in terms of morbidity and third in mortality of cancers (1). Aberrant metabolism, a major hallmark of cancer driven by metabolic reprogramming, is closely linked to GC initiation, progression, and drug resistance, and cancer stem cells (2–4). Many oncogenic signaling pathways, such as Hippo, Myc, and the receptor tyrosine kinase/phosphoinositide 3-kinase/Akt1 cascade promote metabolic gene expression and improve the activity of metabolic enzymes. Conversely, select metabolites not only serve as substrates for energy and biomass generation but can also act as potent signaling modulators by an epigenetic mechanism and even regulate protein production directly (2, 5, 6).

In the past 20 years, the development and application of modern experimental technologies and next-generation sequencing have uncovered not only the metabolic heterogeneity and plasticity of cancers but also novel metabolic signaling involved in cancer biology. Specifically, the extracellular tumor microenvironment (TME), with the depletion of certain nutrients, forces cancer cells to sustain themselves and their progression by inducing a diverse set of metabolic adaptations (6). There is growing appreciation that the metabolism of the stromal cells within the TME, such as endothelial cells, adipocyte, fibroblasts, and myeloid derived suppressor cells, can mediate cancer development (2, 6, 7).

Aberrant metabolism is also a major feature of some inherited human disorders with an inborn error in metabolic pathways. There is increasing evidence regarding the association between congenital metabolic errors and increased risk of cancer development. For example, hyperhomocysteinemia/homocystinuria, which is characterized by an increased level of toxic homocysteine in the plasma due to an inborn error in the metabolic pathways of sulfur-containing amino acids, has close clinical ties with various cancer types (8). Gaucher disease, characterized by enlargement of the internal organs owing to lysosomal storage defection caused by a congenital enzyme acid β-glucosidase deficiency, is strongly correlated with different types of cancers (9). Although the clinical phenotype of these hereditary metabolic diseases is an indicator of some cancers, the relationship between cancer and the genes participating in congenital metabolic errors remains unclear. In addition, few studies have focused on the metabolism of cancer cells themselves rather than the entire TME, including stromal cells.

In this study, we screened hub genes in hereditary metabolic diseases to construct an abnormal metabolism score (AMscore) for both GC diagnosis and prognosis or therapeutic prediction. The diagnostic AMscore displayed excellent sensitivity and specificity in discriminating between GC and normal tissues. The prognostic or predictive AMscore was a strong indicator of both prognosis and the benefit of adjuvant chemotherapy. Moreover, this AMscore was associated with the TME and could efficiently predict the therapy response and survival outcomes of immunotherapy using immune checkpoint inhibitors (ICIs).

## 2 Methods

### 2.1 Genes

Two hundred three hereditary diseases characterized by metabolic abnormalities were identified in the Human Phenotype Ontology database (https://hpo.jax.org/app/; **Supplementary Table S1**). One hundred sixty-two genes, whose aberrant alterations have been verified to cause these diseases, were selected (**Supplementary Table S2**).

### 2.2 GC patients

For diagnostic AMscore construction, GC patients with paired normal and tumor tissues were selected from the Affiliated Hospital of Jiangsu University (AHJU) (10–13), The Cancer Genome Atlas (TCGA) (14), GSE54129 (15), and GSE103236 (16) cohorts (**Supplementary Table S3**). For the prognostic or predictive AMscore construction, GC patients were selected from the Asian Cancer Research Group (ACRG) (17), AHJU (10–13), TCGA (14), GSE15459 (18), and GSE84437 (19) cohorts (**Supplementary Table S4**). The patient enrollment criteria for all cohorts included the following: 1) pathological diagnosis of normal or GC tissues, 2) available transcriptome data, and 3) no prior history of anticancer therapies before sampling. The ethics committee of AHJU approved the research protocol, and all patients from AHJU provided written informed consent.

### 2.3 Immunotherapy patients

Four immunotherapy cohorts were used (**Supplementary Table S5**), including a cohort of metastatic GC (NCT.02589496) treated with second-line pembrolizumab (20), a cohort of metastatic urothelial cancer (UTC; IMvigor210) treated with second-line atezolizumab (21), a cohort of advanced melanoma (CA209-038 or NCT.01621490) treated with first-line or second-line nivolumab (22), and a cohort of advanced non-small-cell lung cancer (NSCLC; GSE135222) treated with antibodies of programmed cell death receptor-1 (PD-1) or its ligand PD-L1 (23).

### 2.4 Multi-omic data

In the AHJU GC cohort, whole exome sequencing (WES), transcriptome sequencing and T cell receptor (TCR)-β CDR3 sequencing were performed. The corresponding genome data and TCR data were stored in the Genome Sequence Archive for Human (https://ngdc.cncb.ac.cn/gsa-human/) with the identifier of HRA001647. The corresponding transcriptome were stored in the European Genome-phenome Archive (https://ega-archive.org/) with the identifier of EGAD00001004164. Multi-omic data from previously published cohorts were acquired and preprocessed as described elsewhere (24). Classic immune indices, such as microsatellite instability (MSI), tumor mutation burden (TMB), and tumor neoantigen burden (TNB) have been previously defined and determined (10–13).

### 2.5 The diagnostic AMscore construction

Based on a random forest model (25), the importance of the expression of targeted genes in distinguishing between normal and tumor tissues was evaluated in TCGA and validated in AHJU. Important genes with significant differential expression between normal and tumor tissues in both TCGA and AHJU were selected. The receiver operating characteristic (ROC) curve and the area under the ROC curve (AUC) were used to evaluate the diagnostic power of each gene. Based on the optimal threshold of maximum ROC curve values, gene expression was dichotomized into high (1) and low (0) levels. The diagnostic AMscore was constructed by binary logistic regression using the forward selection (conditional) method based on gene expression levels. The formula was as follows:


AMscore = intercept + sum (expression level of each gene ×corresponding regression coefficient)

### 2.6 The prognostic or predictive AMscore construction

In cohorts with survival data, the optimal cutoff value to define high and low gene expression with the most significant survival difference was determined using the *Survminer* R package. Gene expression was converted to either 1 (high) or 0 (low) (26). Genes with significant prognostic roles were evaluated using univariate Cox proportional hazards models. The most powerful prognostic genes were further determined using least absolute shrinkage and selection operator (LASSO) Cox regression models. The AMscore model was constructed based on the corresponding regression coefficients. The formula was as follows:


AMscore = sum (expression level of each gene × corresponding coefficient)

Considering the heterogeneous effects of the same gene by tumor type, different gene signatures have been established for different tumor types. In cohorts without survival data, binary logistic regression was used to construct the AMscore to predict therapy response.

### 2.7 Multi-omic sequencing in the AHJU cohort

WES and transcriptome sequencing in the AHJU cohort have been described previously (10–13). Sequencing of TCR-β CDR3 regions in genomic DNA (gDNA) was performed from 26 tissue samples, on which transcriptome sequencing was also conducted. Briefly, gDNA was cut into 200–250-bp fragments and multiplex primers were used to obtain the maximum coverage of a heterogeneous set of target sequences of the V and J families. Then, 151bp paired-end sequencing was performed using the Illumina HiSeq3000 platform (Illumina, USA). MiXCR55 (https://github.com/milaboratory/mixcr/) was used to identify CDR3 protein sequences. TCR diversity was estimated using the Shannon entropy index, and TCR clonality was defined as 1-Pielou’s evenness.

### 2.8 Multiplex immunofluorescence staining

In the AHJU cohort, mIF staining was performed on eight tissue samples with available transcriptome data, using the PANO 7-plex IHC kit (Panovue, Beijing, China). Primary antibodies against CD8 (CST70306, Cell Signaling Technology, USA), CD56 (CST3576), pan-CK (CST4545), CD68 (BX50031, Biolynx, China), and HLA-DR (ab92511, Abcam, UK) were sequentially applied to FFPE tissue slides. Anti-S100 (ab52642) was used to distinguish between the stroma and epithelial parenchyma (27). Imaging was performed using ‘the Leica Bond RX automated staining instrument and ‘Akoya Vectra Polaris spectral quantitative pathological analysis system. Indica ‘Labs HALO software was used to identify the cell types and determine the density of positively stained cells for different markers in the tumor parenchyma and matrix.

### 2.9 Statistical analysis

For comparisons between groups, χ^2^ test, Fisher’s exact probability test, paired or unpaired *t*-test, and Mann–Whitney *U* test were used as needed. The Kaplan–Meier method and log-rank test were used for survival analysis. The independent prognostic role of the AMscore was determined using multivariate Cox proportional hazard models, with calculations of hazard ratios (HRs) and their 95% confidence intervals (CIs). The ROC and AUC were used to evaluate the predictive power of AMscore for the objective response rate (ORR) of immunotherapy. A two-sided p<0.05 was considered statistically significant. Statistical and drawing tools included R (version 3.6.1), R Bioconductor packages, and SPSS (version 19.0; Chicago, IL, USA).

## 3 Results

### 3.1 Genes in hereditary metabolic diseases are biomarkers for GC diagnosis

**Figure 1:**
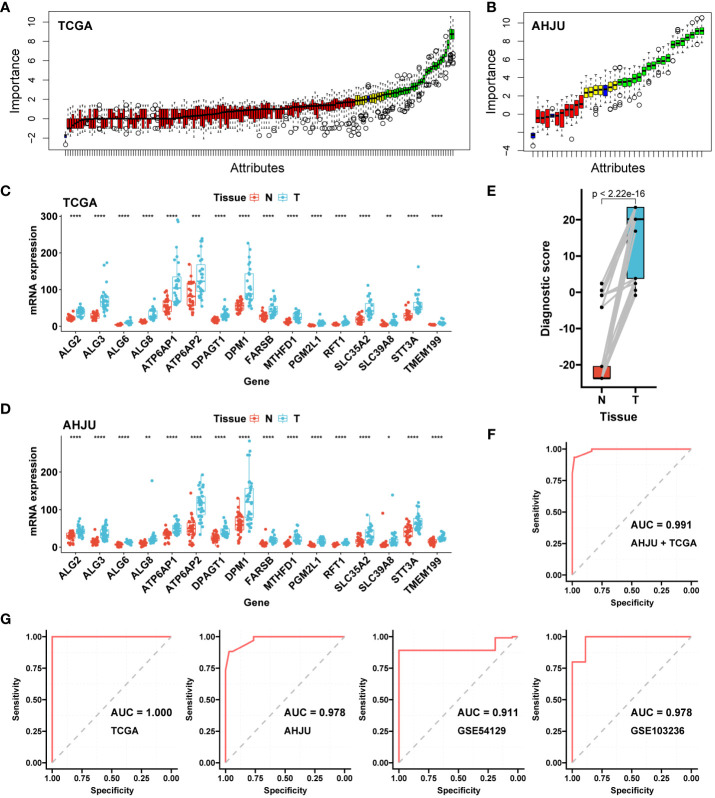
Construction and validation of the diagnostic AMscore. **(A)**: The importance of gene expression associated with abnormal metabolism to distinguish normal and tumor tissues was evaluated by a random forest model in TCGA. **(B)**: Important genes in A were further validated in AHJU. **(C, D)**: The important genes in B with differential expressions (paired t-test) between normal (N) and tumor (T) tissues in both TCGA **(C)** and AHJU **(D)** were selected. **(E)**: Diagnostic score, constructed by a logistic regression based on the expression of genes in **(C, D)**, between normal and tumor tissues (paired t-test). **(F)**: The receiver operating characteristic curve (ROC) of diagnostic score to determine tumor tissues in the combined TCGA and AHJU cohort. **(G)**: The ability of diagnostic score to determine tumor was further validate in TCGA, AHJU, GSE54129 and GSE103236 cohorts, respectively. TCGA, The Cancer Genome Atlas; AHJU, Affiliated Hospital of Jiangsu University; AUC, the areas under the ROC. * p<0.05; ** p<0.01; *** P<0.001; **** p<0.0001.


After random forest screening (**Figures 1A, B**) and differential expression testing (**Figures 1C, D**), 16 genes were used for logistic regression analysis based on the transcriptome data of AHJU and TCGA. Finally, four genes were included in the model to construct the diagnostic AMscore in the combined AHJU and TCGA cohort, using the following formula:

$$\begin{array}{l} Amscore\;=\;-23.74\;+\;21.002\;\times \;expression\;level\;of\;ALG3\;+\;3.26\;\times \;\\ expression\;level\;of\;PGM2L1\;+\;19.603\;\times \;expression\;level\;of\;SLC39A8\;+3.285\;\times \;\\ expression\;level\;of\;TMEM199 \end{array}$$

The AMscore was significantly higher in GC tissues than in paired normal tissues (p<2.22e-16; **Figure 1E**), with an AUC of 0.991 (sensitivity: 0.93, and specificity: 0.98) for predicting GC in the combined AHJU and TCGA cohorts (**Figure 1F**). For validation, the diagnostic AUC of the AMscore was also favorable in separate TCGA (AUC: 1.000, sensitivity: 1.00, and specificity: 1.00), AHJU (AUC: 0.978, sensitivity: 0.88, and specificity: 0.97), GSE54129 (AUC: 0.911, sensitivity: 0.89, and specificity: 1.00), and GSE103236 (AUC: 0.978, sensitivity: 1.00, and specificity: 0.89) cohorts (**Figure 1G**).

### 3.2 Genes in hereditary metabolic diseases are biomarkers for GC prognosis

**Figure 2:**
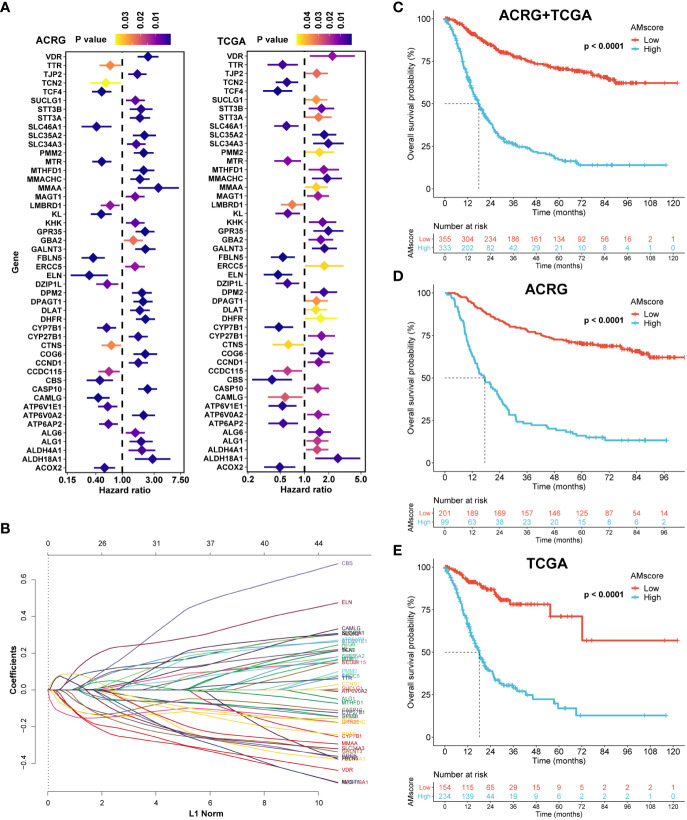
Construction of the prognostic or predictive AMscore. **(A)**: Genes associated with prognosis of gastric cancer in both the ACRG and TCGA cohorts. **(B)**: LASSO coefficient profiles of the fractions of the genes in A in the combined ACRG and TCGA cohort. **(C–E)**: AMscore and overall survival in the combined ACRG and TCGA cohort **(C)** and in the individual ACRG **(D)** and TCGA **(E)** cohorts, respectively. ACRG, Asian Cancer Research Group; TCGA, The Cancer Genome Atlas.

**Figure 3:**
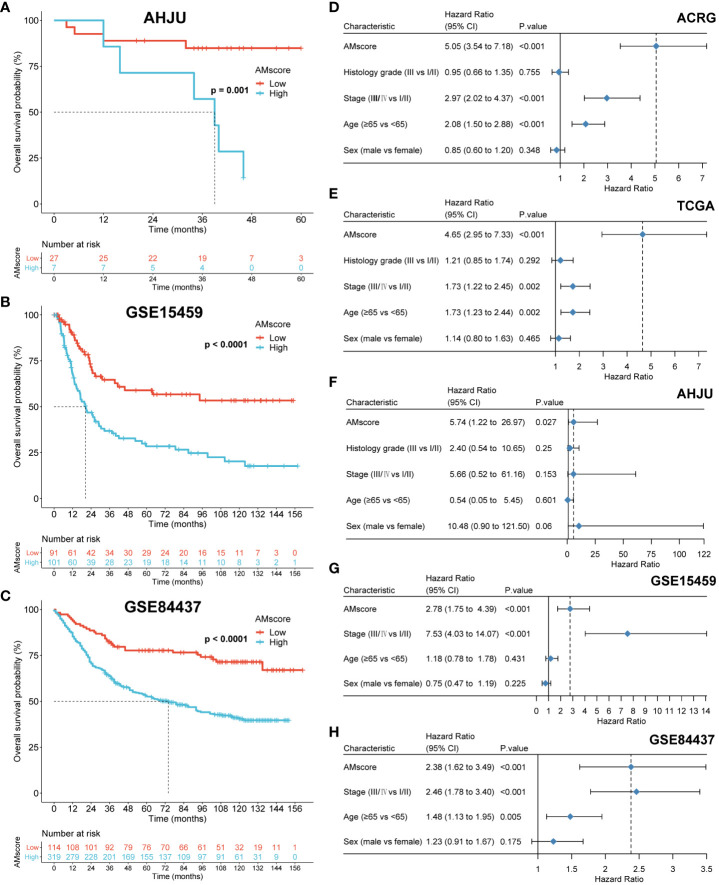
Validation of the prognostic or predictive AMscore. **(A–C)**: The prognostic role of AMscore in the AHJU, GSE15459 and GSE84437 cohorts. **(D–H)**: in multivariate Cox regression models, AMscore was an independent predictor for overall survival in the ACRG **(D)**, TCGA **(E)**, AHJU **(F)**, GSE15459 **(G)** and GSE84437 **(H)** cohorts, respectively. ACRG, Asian Cancer Research Group; AHJU, Affiliated Hospital of Jiangsu University; TCGA, The Cancer Genome Atlas.

After univariate Cox analysis (**Figure 2A**), 48 genes with a significant prognostic impact on overall survival (OS) in both ACRG and TCGA cohorts were included in the LASSO regression model (**Figure 2B**). Finally, 32 genes were included in the model to construct the prognostic or predictive AMscore in the combined ACRG and TCGA cohorts; the corresponding regression coefficients are shown in **Supplementary Table S6**. The OS was significantly shorter in the high AMscore subgroup than in the low AMscore subgroup in the combined ACRG and TCGA cohorts (**Figure 2C**), as well as in the separate ACRG (**Figure 2D**) and TCGA (**Figure 2E**) cohorts. Moreover, the prognostic role of the AMscore was further validated in the AHJU, GSE15459, and GSE84437 cohorts (**Figures 3A–C**). Importantly, the multivariate Cox models showed that the AMscore was an independent predictor of OS in all GC cohorts included in our survival analysis (**Figures 3D–H**).

## 3.3 Genes in hereditary metabolic diseases involve broad biological processes

**Figure 4:**
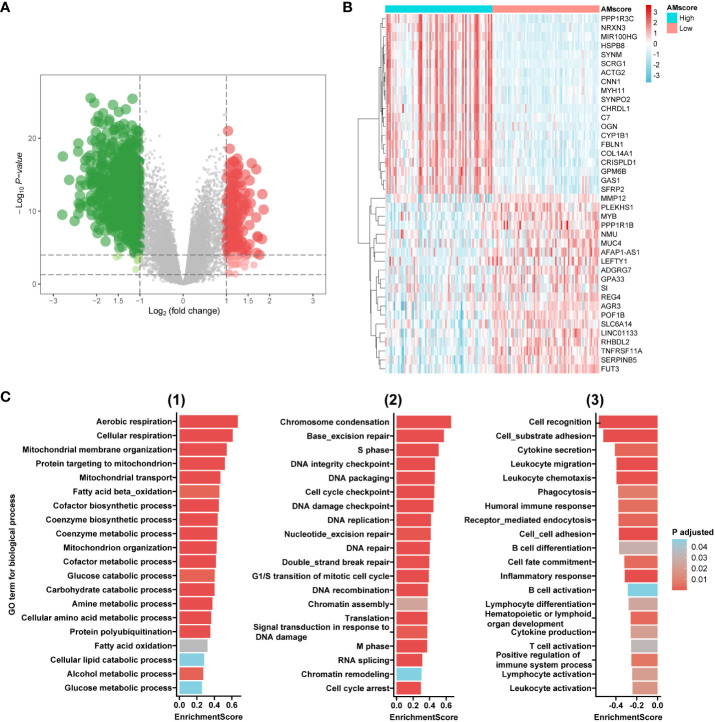
Transcriptome features associated with the prognostic or predictive AMscore. **(A)**: Volcano plot for differentially expressed genes (DEGs) between high and low AMscore subgroups. **(B)**: Heatmap for top 40 DEGs between high and low AMscore subgroups. **(C)**: Selected Gene Ontology (GO) terms for biological process in the gene set enrichment analysis of DEGs (1): metabolism-associated terms (2); DNA repair- and cell cycle-associated terms (3): immunity-associated terms.

In the ACRG cohort, differentially expressed genes were identified between the high (upper quartile) and low (lower quartile) subgroups of the prognostic or predictive AMscore, based on the criteria of adjusted p-value<0.05, and log2(fold change)>1 (**Figures 4A, B**). Gene set enrichment analysis (GSEA) was performed using NetworkAnalyst 3.0 (https://www.networkanalyst.ca/) based on Gene Ontology (GO) terms of biological processes. We found that the enriched GO terms in the high AMscore group could be divided into three main sections (1): metabolic processes involving glucose, fats, amino acids, proteins, and others (2); cell proliferation involving DNA replication, DNA damage response and repair, the cell cycle, and so on (3); immune activities involving immune organ development, immune cell differentiation and activation, immune response, and so on (**Figures 4C**; **Supplementary Table S7**). These results suggest that tumors with high AMscore have a growth and survival advantage in the TME of GC.

### 3.4 The prognostic or predictive AMscore and the benefit of postoperative adjuvant therapy in GC

**Figure 5:**
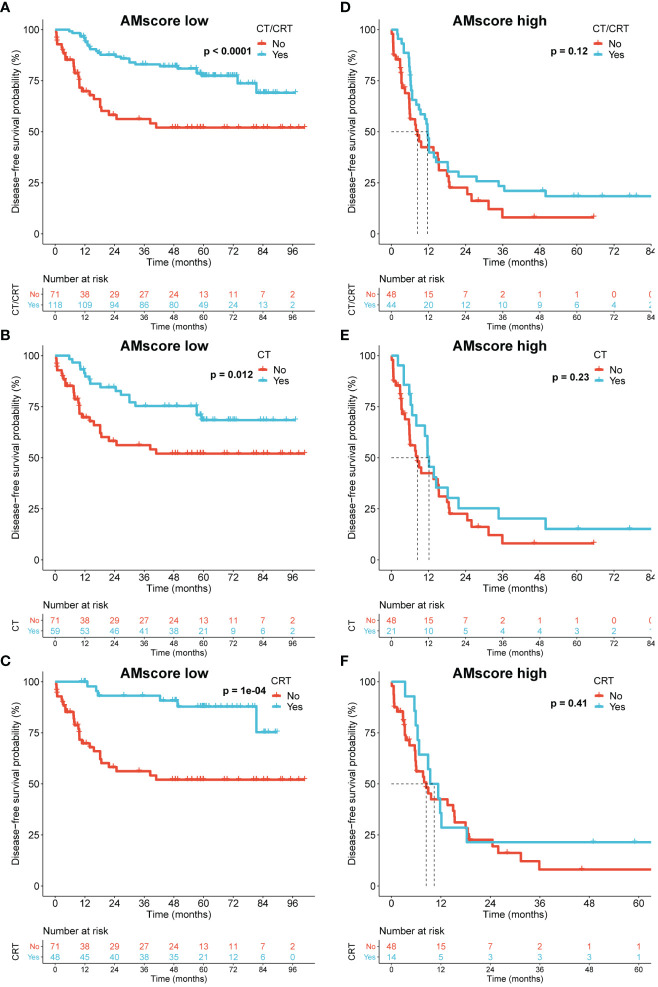
The prognostic or predictive AMscore and the benefit of adjuvant chemotherapy or chemoradiotherapy in the ACRG cohort. **(A–C)**: Disease-free survival (DFS) benefit was significant in the AMscore low subgroup for CT/CRT **(A)**, CT **(B)**, and CRT **(C)**, respectively. **(D–F)**: DFS benefit was not significant in the AMscore high subgroup for CT/CRT **(D)**, CT **(E)**, and CRT **(F)**, respectively. ACRG, Asian Cancer Research Group; CT, chemotherapy; CRT, chemoradiotherapy.

Because our GSEA indicated an association between the AMscore and DNA repair, a crucial mechanism by which tumor cells resist chemotherapy (CT) or chemoradiotherapy (CRT) (28), we investigated the impact of the AMscore on the benefit of postoperative CT/CRT in GC in the ACRG cohort, which has detailed treatment information. In patients treated with postoperative CT/CRT, CT, or CRT, the AMscore remained a strong prognostic predictor for both disease-free survival (DFS) and OS (**Supplementary Figure S1**). Importantly, adjuvant CT/CRT, CT, or CRT significantly improved DFS in patients with a low AMscore but not in those with a high AMscore (**Figure 5**). Similar results were observed in patients with a low AMscore, and substantially decreased benefits for CT (p=0.089), CRT (p=0.095), and CT/CRT (p=0.014) were observed in patients with a high AMscore (**Supplementary Figure S2**).

### 3.5 The prognostic or predictive AMscore and classic immune biomarkers

**Figure 6:**
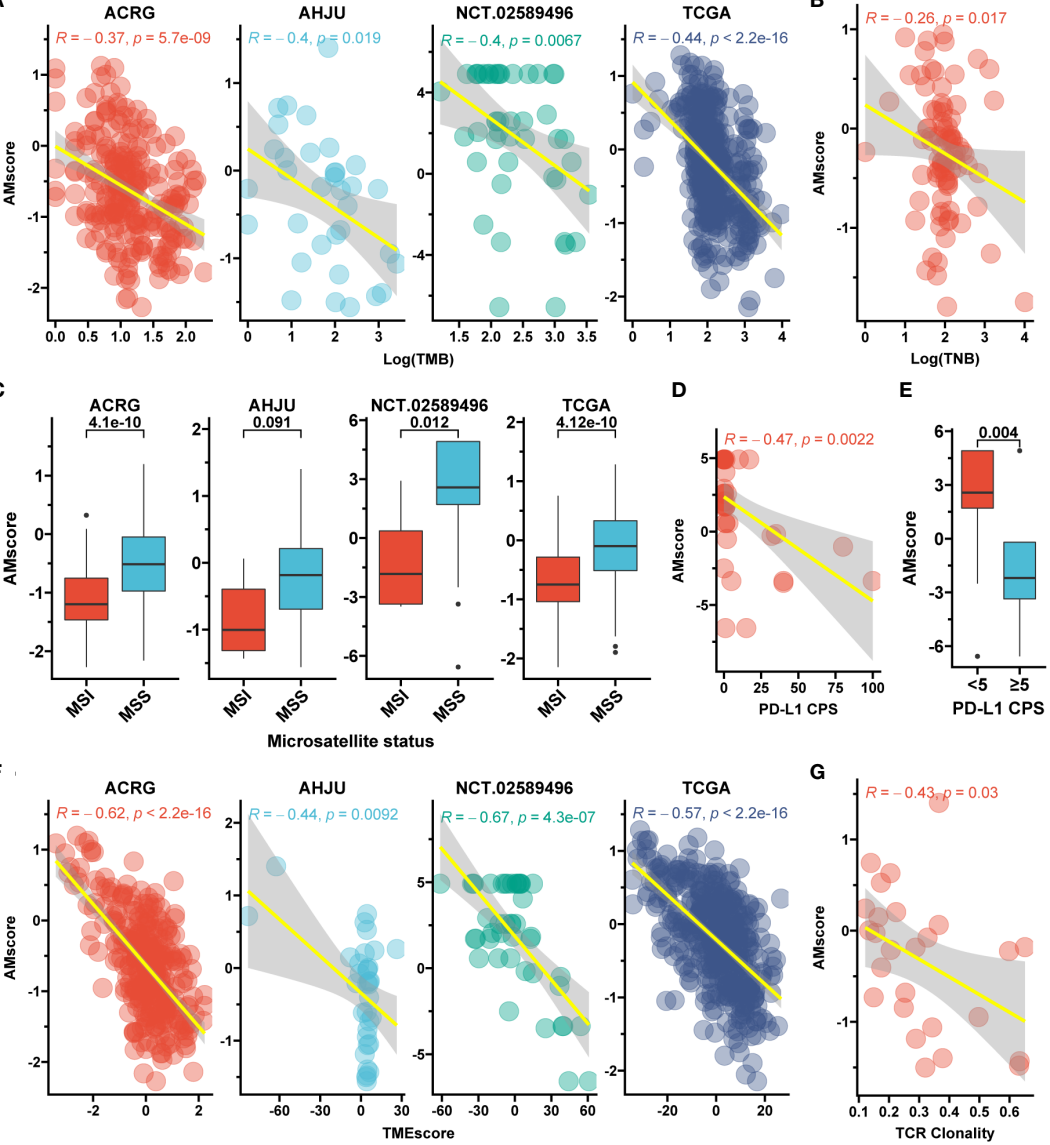
The prognostic or predictive AMscore and immune biomarkers. **(A)**: Correlation between AMscore and tumor mutation burden (TMB). **(B)**: Correlation between AMscore and tumor neoantigen burden (TNB) in the TCGA cohort. **(C)**: AMscore according to microsatellite status; **(D)**: Correlation between AMscore and PD-L1 combined positive score (CPS). **(E)**: AMscore according to PD-L1 CPS level. **(F)**: Correlation between AMscore and TMEscore. **(G)**: Correlation between AMscore and T cell receptor (TCR) clonality in the AHJU cohort. ACRG, Asian Cancer Research Group; AHJU, Affiliated Hospital of Jiangsu University; TCGA, The Cancer Genome Atlas. MSI, microsatellite instability; MSS, microsatellite stability.

Given the potential influence of AMscore on immune activity indicated by our GSEA, the relationship between AMscore and classic immune biomarkers was explored. A significantly negative correlation was found between the AMscore and TMB in the ACRG, AHJU, NCT.02589496, and TCGA cohorts (**Figure 6A**). TNB was detected in 84 samples from TCGA and was also negatively correlated with the AMscore (**Figure 6B**). Regarding the microsatellite status, the MSI subtype of GC had a significantly lower AMscore than the microsatellite stable (MSS) subtype in all cohorts (**Figure 6C**). In the NCT.02589496 cohort, PD-L1 expression determined using a combined positive score (CPS) was detected by immunohistochemistry. The AMscore was negatively correlated with PD-L1 CPS (r=-0.47, p=0.002; **Figure 6D**), and a significantly lower AMscore was observed in the subgroup with a CPS≥5 (**Figure 6E**). Moreover, the TMEscore, an index previously developed to evaluate the TME of GC (24), was also calculated to show a significantly negative correlation with the AMscore in all cohorts (**Figure 6F**). Finally, TCR sequencing was conducted in the AHJU cohort, and high TCR clonality indicated superior clonal expansion of TCR and a potentially strong immune response (29). The AMscore was also negatively correlated with TCR clonality (**Figure 6G**).

### 3.6 The prognostic or predictive AMscore and immune cell infiltration

**Figure 7:**
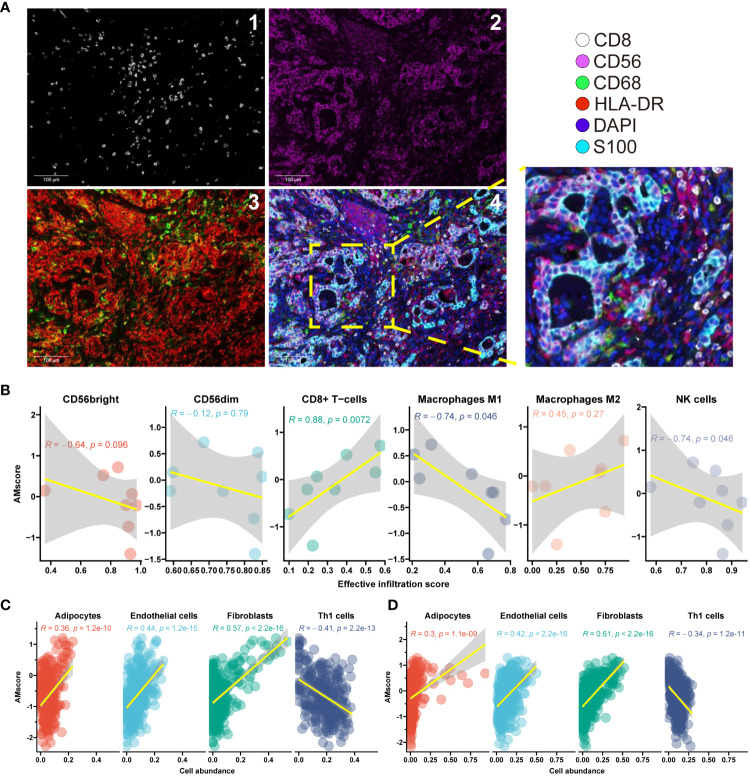
The prognostic or predictive AMscore and immune cell infiltration. **(A)**: Typical photomicrograph for multiple-immunofluorescence staining of surface markers of immune cells in the AHJU cohort. 1: CD8; 2: CD56; 3: HLA-DR (red) and CD68 (green); and 4: reconstructed image including all markers. **(B)**: Correlation between AMscore and the effective infiltration scores of immune cells in the AHJU cohort. **(C, D)**: Correlation between AMscore and the abundances of other cells estimated based on transcriptome in the ACRG **(C)** and TCGA **(D)** cohorts.

mIF was performed in the AHJU cohort to quantify the density of infiltrating immune cells in the TME (**Figure 7A**). The effective infiltration score (EIS), defined as the number of immune cells in the tumor parenchyma divided by the total number of immune cells in TME, was used to evaluate the mobilization of immune cells from the stromal tumor edge into the tumor parenchyma, which is crucial for antitumor immunity (12, 13, 27). The AMscore was significantly positively correlated with the EIS of CD8+ T cells (Spearman r=0.88, p=0.007) but significantly negatively correlated with the EIS of M1 macrophages (r=-0.74, p=0.046) and NK cells (r=-0.74, p=0.046). The AMscore was also correlated with the EIS of M2 macrophages (r=0.45, p=0.27) and the CD56bright subtype of NK cells (r=-0.64, p=0.096), and the significance of these results may be limited by the small sample size (**Figure 7B**). The xCell algorithm (30) was used to evaluate the abundance of other TME cells based on the transcriptome data of the ACRG and TCGA. The AMscore was also significantly positively correlated with the abundance of adipocytes, endothelial cells, and fibroblasts, but significantly negatively correlated with the abundance of Th1 cells (**Figures 7C, D**).

### 3.7 The prognostic or predictive AMscore and immunotherapy outcomes

**Figure 8:**
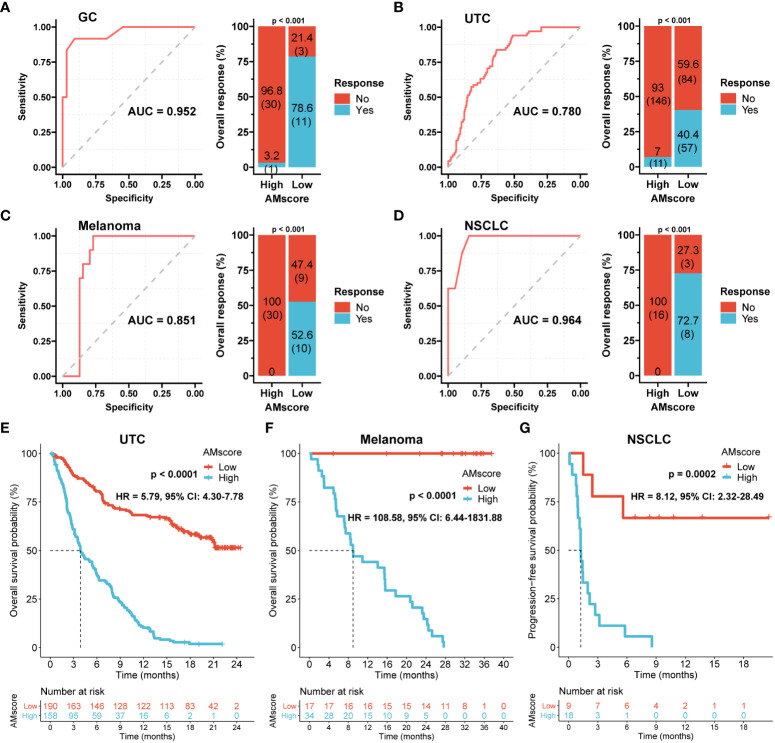
The prognostic or predictive AMscore and immunotherapy outcomes. **(A–D)**: The receiver operating characteristic curve (ROC) for response prediction and the objective response rate by AMscore in the immunotherapeutic gastric cancer (GC; **A**), urothelial cancer (UTC; **B**), melanoma **(C)**, and non-small-cell lung cancer (NSCLC; **D**) cohorts, respectively. **(E–G)**: AMscore level significantly stratified overall survival in the immunotherapeutic UTC **(E)** and melanoma **(F)** cohorts, and progression-free survival in the NSCLC cohort **(G).** AUC: the areas under the ROC.

In the immunotherapy cohorts of GC, UTC, melanoma, and NSCLC, the AUCs of the AMscore for predicting therapy response were 0.952, 0.780, 0.851, and 0.964, respectively (**Figures 8A–D**), which were generally better than those of classic biomarkers (**Supplementary Figure S3**). The ORRs of low versus high AMscore in these four cohorts were 78.6% vs. 3.2%, 40.4% vs. 7.0%, 52.6% vs. 0%, and 72.7% vs. 0%, respectively (all p<0.001; **Figures 8A–D**). OS was available in the UTC and melanoma cohorts, and patients with a high AMscore presented significantly shorter OS than those with a low AMscore in both cohorts (HR = 5.79, 95% CI: 4.30-7.78, and HR = 108.58, 95% CI: 6.44-1831.88, respectively, both p<0.0001; **Figures 8E, F**). A similar result was found for progression-free survival (PFS) in the NSCLC cohort (HR = 8.12, 95% CI: 2.32-28.49, p=0.0002; **Figure 8G**). In the melanoma cohort, subgroup analysis showed that the ORRs of low versus high AMscore were 66.7% vs. 0% and 40% vs. 0% for the first-line and second-line immunotherapy, respectively (**Supplementary Figures S4A, B**). And significant OS superiority still were observed for patients with a low AMscore regardless of treatment lines (**Supplementary Figures S4C, D**).

The combined role of the AMscore and classic immunotherapy biomarkers was investigated (**Table 1**). In all the MSI, high TMB, and PD-L1 CPS≥5 subsets of the GC cohort, patients with a low AMscore showed an ORR of 100%, and those with a high AMscore had an ORR of 0%. A low AMscore still displayed significant superiority over a high AMscore in terms of ORR, even in the MSS (66.7% vs. 3.3%, p<0.001), low TMB (50.0% vs. 3.7%, p=0.002), and PD-L1 CPS<5 (50.0% vs. 0%, p<0.001) subsets. This huge response advantage in the low-AMscore subgroup, independent of other biomarkers, was further validated in the UTC, melanoma, and NSCLC cohorts, regardless of stratification according to TMB, TNB, PD-L1 expression on tumor cells, PD-L1 expression on immune cells (IC), or immune phenotype. Moreover, a huge survival advantage in the low AMscore subgroup was observed in all cohorts in all stratifications according to classic biomarkers (**Supplementary Figures S5**, **S6**).

**Table 1:** Immunotherapy response by the prognostic or predictive AMscore stratified by classic immune biomarkers.

Biomarker	Response	AMscore		P value
		Low (%)	High (%)	
In the GC cohort (NCT.02589496)				
Microsatellite status				
MSS	No	3 (33.3)	29 (96.7)	<0.001
	Yes	6 (66.7)	1 (3.3)	
MSI	No	0 (0.0)	1 (100.0)	0.014
	Yes	5 (100.0)	0 (0.0)	
Tumor mutation burden^*^				
Low	No	3 (50.0)	26 (96.3)	0.002
	Yes	3 (50.0)	1 (3.7)	
High	No	0 (0.0)	4 (100.0)	0.001
	Yes	8 (100.0)	0 (0.0)	
PD-L1 combined positive score (CPS)				
<5	No	3 (50.0)	25 (100.0)	<0.001
	Yes	3 (50.0)	0 (0.0)	
≥5	No	0 (0.0)	2 (100.0)	0.002
	Yes	8 (100.0)	0 (0.0)	
In the urothelial cancer cohort (IMvigor210)				
Tumor mutation burden^*^				
Low	No	70 (70.7)	113 (94.2)	<0.001
	Yes	29 (29.3)	7 (5.8)	
High	No	14 (33.3)	33 (89.2)	<0.001
	Yes	28 (66.7)	4 (10.8)	
Tumor neoantigen burden^*^				
Low	No	71 (71.7)	132 (95.0)	<0.001
	Yes	28 (28.3)	7 (5.0)	
High	No	13 (31.0)	14 (77.8)	0.001
	Yes	29 (69.0)	4 (22.2)	
PD-L1 expression on immune cells (IC)				
IC0	No	21 (65.6)	49 (96.1)	<0.001
	Yes	11 (34.4)	2 (3.9)	
IC1	No	32 (65.3)	60 (95.2)	<0.001
	Yes	17 (34.7)	3 (4.8)	
IC2	No	30 (50.8)	37 (86.0)	<0.001
	Yes	29 (49.2)	6 (14.0)	
PD-L1 expression on tumor cells (TC)				
TC0	No	67 (60.4)	118 (92.9)	<0.001
	Yes	44 (39.6)	9 (7.1)	
TC1	No	4 (44.4)	8 (100.0)	0.012
	Yes	5 (55.6)	5 (0.0)	
TC2	No	12 (60.0)	20 (90.9)	0.019
	Yes	8 (40.0)	2 (9.1)	
Immune phenotype				
Inflamed	No	20 (52.6)	23 (95.8)	<0.001
	Yes	18 (47.4)	1 (4.2)	
Desert	No	15 (62.5)	40 (88.9)	0.014
	Yes	9 (37.5)	5 (11.1)	
Excluded	No	30 (56.6)	55 (91.7)	<0.001
	Yes	23 (43.4)	5 (8.3)	
In the melanoma cohort (NCT.01621490)				
Tumor mutation burden^*^				
Low	No	7 (53.8)	14 (100.0)	0.006
	Yes	6 (46.2)	0 (0.0)	
High	No	2 (33.3)	11 (100.0)	0.006
	Yes	4 (66.7)	0 (0.0)	
Tumor neoantigen burden^*^				
Low	No	4 (80.0)	–	
	Yes	1 (20.0)	–	
High	No	5 (35.7)	25 (100.0)	<0.001
	Yes	9 (64.3)	0 (0.0)	
In the NSCLC cohort (GSE135222)				
Tumor mutation burden^*^				
Low	No	2 (33.3)	16 (94.1)	0.002
	Yes	4 (66.7)	1 (5.9)	
High	No	0 (0.0)	1 (100.0)	0.046
	Yes	3 (100.0)	0 (0.0)	

^*^Based on the optimal threshold of TMB/TNB for the maximum ROC curve values, the patients were dichotomized into high and low subgroups.

GC, gastric cancer; NSCLC, non-small-cell lung cancer.

## 4 Discussion

Abnormal metabolism is a common phenotype in cancers and hereditary diseases. Genes responsible for inborn metabolic errors may also play important roles in cancer development. However, in contrast to abnormal hereditary metabolism, which is usually caused by defects in a single or a few genes (**Table S1**), cancer metabolism is more complicated and involves polygenes and various metabolic pathways, which require a comprehensive evaluation based on omics. Thus, this study established gene signatures and generated corresponding scores to evaluate the overall role of genes involved in hereditary metabolic diseases in the diagnosis, prognosis, and treatment outcomes of GC.

In this study, a diagnostic signature and a prognostic or predictive signature were established. Interestingly, these two signatures were completely different. The potential explanations included: genes play disparate roles during different phases of cancer (31); carcinogenesis and cancer progression may be driven by different genes (32); metabolic genes play a dynamic role in cancer through metabolic reprogramming (2). Furthermore, only 4 genes in the diagnostic signature and up to 32 genes in the prognostic or predictive signature indicated that an increasing number of metabolic genes were gradually activated during cancer development.

Our diagnostic AMscore robustly distinguished between GC and normal tissues in the different cohorts. In its signature, both ALG3 and SLC39A8 play central roles because of their large regression coefficients. Of these, *ALG3*, encoding alpha-1,3-mannosyltransferase, is associated with nitrogen-linked glycosylation, which regulates various cellular processes, including cell recognition, signal transduction, and cell-matrix interactions (33). Defects in ALG3 have been associated with a congenital disorder of glycosylation type Id characterized by severe neurological involvement (34). Recently, increasing evidence has revealed that ALG3 overexpression promotes carcinogenesis, tumor proliferation, metastasis, and radio resistance and impairs antitumor immunity in several cancers (35–38). The other gene, SLC39A8, encodes the zinc ion transporter ZIP8, which mediates the cellular uptake of divalent metal ions, including zinc, iron, manganese, and cadmium, and is therefore essential for the growth, development, and normal function of tissues and organs (39). Interestingly, SLC39A8 defects are associated with another congenital disorder, glycosylation type IIn (40). Recently, abnormal SLC39A8 expression has been reported to increase cancer risk and impact the clinical outcomes of several cancers, partly through ferroptosis-related mechanisms (41–43).

In the model of prognostic or predictive AMscore, many genes presented an appreciable coefficient, indicating a joint metabolic effect on clinical outcomes. *CBS*, the largest coefficient contributor, encodes cystathionine beta-synthase, which catalyzes the conversion of homocysteine to cystathionine, a critical step in the generation of hydrogen sulfide (H2S). Defects in this gene can cause homocystinuria due to cystathionine beta-synthase deficiency (44). Because of the close relationship between homocystinuria and cancer, CBS plays a significant pathogenetic role in cancer and is linked to metastasis and multidrug resistance owing to the important regulatory effect of H2S on mammalian biology, physiology, and pathophysiology (45). However, contradictory findings have been reported in different cancers, indicating a cancer type-dependent role of CBS (45).

Our prognostic or predictive signature involved many metabolic activities that contributed to various biological processes, especially those associated with therapy resistance and anticancer immunity, as suggested in the GSEA according to AMscore levels. Consistent with these results, we further found that the prognostic or predictive AMscore determined the benefit of postoperative adjuvant CT/CRT in GC, which lacks biomarkers, and therefore has the potential to improve patient selection for this therapy. We also revealed that the AMscore had a negative impact on TMB, TNB, and MSI, together with a significant enrichment of DNA repair signaling in the high AMscore group in GSEA, suggesting that tumors with a high AMscore are genome-stable. In addition, a significant negative correlation was observed between the AMscore and PD-L1 CPS. Energy status and specific metabolic pathways have been verified to dictate PD-L1 protein levels (46, 47). Furthermore, the AMscore was also correlated with immune cell infiltration, and a high AMscore indicated impaired anticancer immunity, characterized by decreased EIS of M1 macrophages and NK cells. Interestingly, a high AMscore correlated with a high EIS of CD8+ T cells, indicating the aberrant metabolism of this critical immune cell. Recent studies have focused on targeting T cell metabolism to unleash T cell activity (48). In addition, the metabolism of stromal cells in the TME plays a pivotal role in tumor progression and maintenance (49). We also showed that the AMscore positively correlated with stromal cells such as adipocytes, endothelial cells, and fibroblasts.

Recently, immunotherapy represented by ICIs has been a major breakthrough in GC therapy. In the first-line treatment of metastatic GC, five pivotal phase III trials, CheckMate 649, ATTRACTION-4, ORIENT-16, RATIONALE305, and KEYNOTE-859, showed that the combination of ICIs with chemotherapy significantly prolonged PFS and/or OS compared to chemotherapy alone in patients with positive or high PD-L1 expression and in the entire population (50). However, the survival benefit of immunotherapy is still disputed in patients with low or negative PD-L1 expression, which was not found in a *post-hoc* analysis (51). In some trials, such as the CheckMate 649 (52) and ORIENT-16 (53), the survival benefit in the entire population appeared to mainly come from patients with high PD-L1 expression, whose proportions were unusually high (60% and 61%, respectively). Although PD-L1 seems to be an appropriate predictor of immunotherapy, PD-L1 alone is not sufficient to meet the increasing pursuit of therapeutic efficacy. Therefore, other classic biomarkers, such as TMB and MSI, are also used for clinical decisions. However, these current biomarkers have limitations, including a lack of standard testing, the impact of intra-tumor heterogeneity, inconsistent efficacy association between trials and cancers, and applicability limited to a minority (53). Novel biomarkers are needed to improve existing strategies for patient selection to increase therapeutic efficacy and decrease ineffective treatment. In this study, the AMscore was shown to be a robust predictor of both immunotherapy response and survival in GC and other tumors. Importantly, the AMscore improved the predictive capability of the current biomarkers. In particular, an ORR of 100% was observed in patients with a co-occurrence of low AMscore and MSI, high TMB or PD-L1 CPS≥5 in the GC and NSCLC cohorts included in our study. More notably, the AMscore screened out patients with favorable treatment outcomes from the disadvantaged groups defined by current biomarkers, thus redefining the advantaged and disadvantaged groups. These promising results demonstrate the close relationship between metabolism, immunity, and the effectiveness of immunotherapy.

This study had some limitations. First, many genes in our signatures have unclear roles in cancer biology, although they provide novel targets for further research. Second, the specificity of our diagnostic AMscore to separate GC from other cancers was not investigated in this study, which is still a challenge faced by existing diagnostic biomarkers. Third, only the ACRG cohort with sufficient data was used to investigate the association of the AMscore with the adjuvant therapy benefit of GC; more such cohorts are needed. Moreover, a few patients in the cohorts included in this study received first-line immunotherapy, especially in combination with chemotherapy, which has been the standard treatment for GC and some other cancers. Besides, the stronger combination of chemotherapy, targeted therapy, and immunotherapy has been tested in GC (50). Therefore, the predictive role of the AMscore needs investigated in these therapies. Finally, our results require prospective validation.

In conclusion, this study revealed a strong association of genes in hereditary metabolic diseases with the diagnosis, prognosis, and therapeutic outcomes of GC and showed the potential for the use of related gene signatures and corresponding scoring to guide clinical practice. Further validation is necessary and future research should focus on specific hub genes.

## Data availability statement

The datasets presented in this study can be found in online repositories. The names of the repository/repositories and accession number(s) can be found below: The corresponding genome data and TCR data were stored in the Genome Sequence Archive for Human (https://ngdc.cncb.ac.cn/gsa-human/) with the identifier of HRA001647. The corresponding transcriptome were stored in the European Genome-phenome Archive (https://ega-archive.org/) with the identifier of EGAD00001004164.

## Ethics statement

The studies involving humans were approved by the ethics committee of Affiliated Hospital of Jiangsu University. The studies were conducted in accordance with the local legislation and institutional requirements. The participants provided their written informed consent to participate in this study.

## Author contributions

YL: Conceptualization, Formal Analysis, Investigation, Methodology, Resources, Software, Validation, Writing – original draft. XL: Conceptualization, Investigation, Methodology, Resources, Validation, Writing – original draft, Data curation. YY: Conceptualization, Data curation, Investigation, Methodology, Validation, Formal Analysis, Writing – original draft. XQ: Data curation, Formal Analysis, Investigation, Methodology, Writing – original draft. QT: Data curation, Formal Analysis, Investigation, Methodology, Writing – original draft. CP: Data curation, Formal Analysis, Investigation, Writing – original draft. MH: Data curation, Formal Analysis, Investigation, Writing – review & editing. KD: Data curation, Investigation, Writing – review & editing. MX: Investigation, Conceptualization, Formal Analysis, Methodology, Project administration, Resources, Supervision, Validation, Writing – review & editing. DW: Conceptualization, Formal Analysis, Investigation, Methodology, Project administration, Resources, Supervision, Validation, Writing – original draft, Writing – review & editing, Data curation, Funding acquisition, Software, Visualization. GH: Conceptualization, Data curation, Formal Analysis, Funding acquisition, Investigation, Methodology, Project administration, Resources, Software, Supervision, Validation, Visualization, Writing – review & editing.

## Supplementary material

Supplementary Figure 1 The prognostic or predictive AMscore and prognosis of patients treated by adjuvant chemotherapy or chemoradiotherapy. **(A–C)**: Overall survival stratified by AMscore levels in patients treated by adjuvant CT/RCT **(A)**, CT **(B)** or RT **(C)**, respectively. **(D–F)**: Disease-free survival stratified by AMscore levels in patients treated by adjuvant CT/RCT **(D)**, CT **(E)** or RT **(F)**, respectively. CT: chemotherapy; CRT: chemoradiotherapy.

Supplementary Figure 2 The prognostic or predictive AMscore and the overall survival benefit of adjuvant chemotherapy or chemoradiotherapy in the ACRG cohort. **(A–C)**: Overall survival (OS) benefit was significant in the AMscore low subgroup for CT/CRT **(A)**, CT **(B)**, and CRT **(C)**, respectively. **(D–F)**: Compared with the results in the AMscore low subgroup, OS benefit was substantially decreased in the AMscore high subgroup for CT/CRT **(D)**, CT **(E)**, and CRT **(F)**, respectively. ACRG: Asian Cancer Research Group; CT: chemotherapy; CRT: chemoradiotherapy.

Supplementary Figure 3 Immunotherapy response prediction by classic biomarkers. **(A–D)**: The receiver operating characteristic curve for response prediction by classic biomarkers in the immunotherapeutic gastric cancer **(A)**, urothelial cancer **(B)**, melanoma **(C)**, and non-small-cell lung cancer **(D)** cohorts, respectively. TMB: tumor mutation burden; TNB: tumor neoantigen burden; MSI: microsatellite instability; CPS: combined positive score; IP: immune phenotype; IC: immune cells; TC: tumor cells.

Supplementary Figure 4 Subgroup analysis in the melanoma cohort. **(A, B)**: Overall response rates according to AMscore in the first-line **(A)** and second-line immunotherapy **(B)**. **(C, D)**: Overall survival according to AMscore in the first-line **(C)** and second-line immunotherapy **(D)**.

Supplementary Figure 5 Overall survival of immunotherapy stratified by the prognostic or predictive AMscore according to TMB or TNB levels. **(A, B)**: the gastric cancer cohort; **(C–E)**: the urothelial cancer **(C)**, melanoma (**D**; all TNB low samples were also AMscore low), and non-small-cell lung cancer **(E)** cohorts, respectively. TMB: tumor mutation burden; TNB: tumor neoantigen burden.

Supplementary Figure 6 Overall survival of immunotherapy stratified by the prognostic or predictive AMscore according to immune phenotype and PD-L1 expression in urothelial cancer. **(A)**: immune phenotype. **(B)**: PD-L1 expression on immune cells (IC). **(C)**: PD-L1 expression on tumor cells (TC).
