# Supplementary figures and images for: Association of genes in hereditary metabolic diseases with diagnosis, prognosis, and treatment outcomes in gastric cancer
Source: Front Immunol. 2023 Nov 9;14:1289700. doi: 10.3389/fimmu.2023.1289700 (PMC10665511; doi:10.3389/fimmu.2023.1289700)

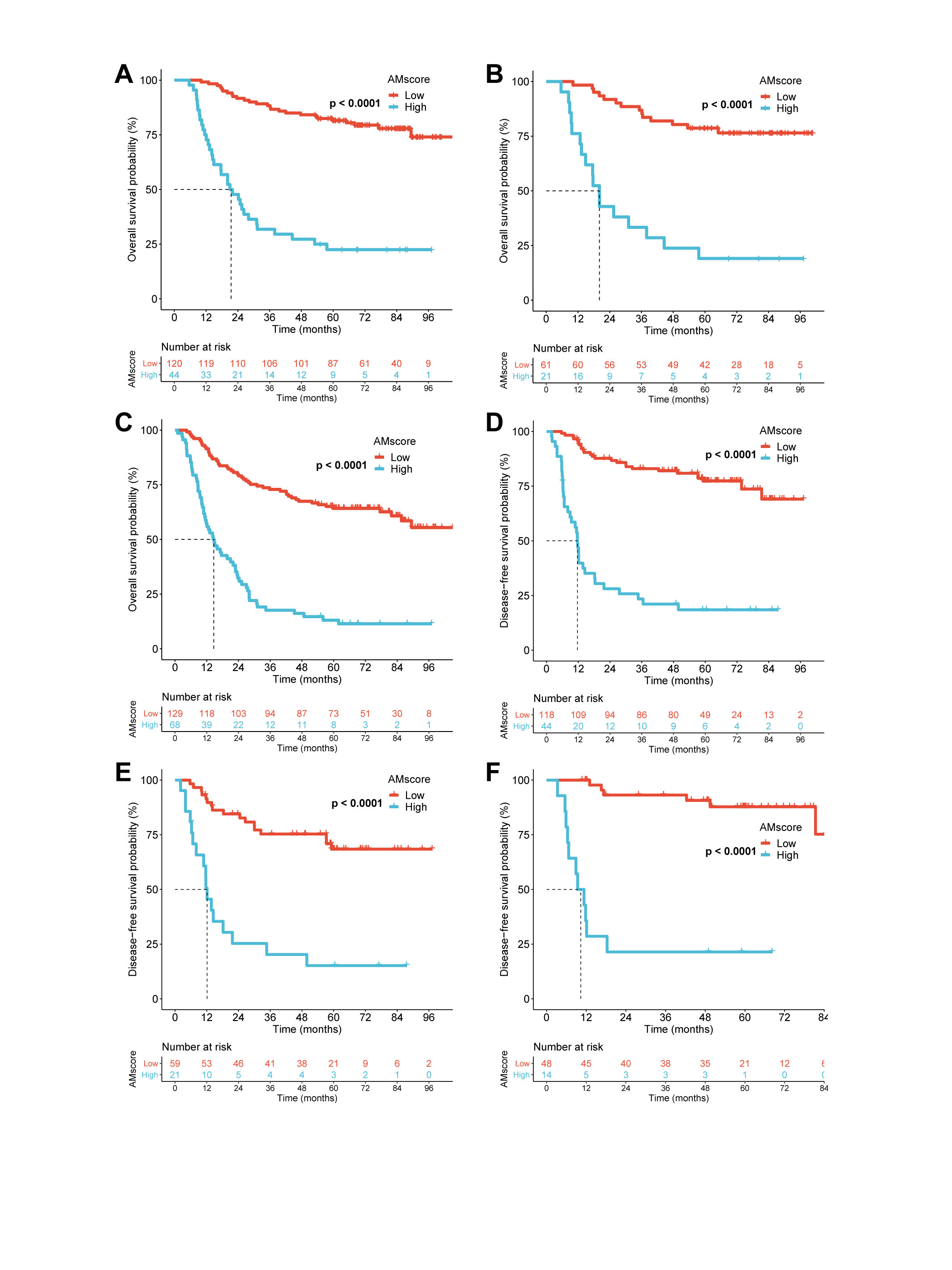

Supplement: Supplementary Figure 1 — The prognostic or predictive AMscore and prognosis of patients treated by adjuvant chemotherapy or chemoradiotherapy. (A–C): Overall survival stratified by AMscore levels in patients treated by adjuvant CT/RCT (A), CT (B) or RT (C), respectively. (D–F): Disease-free survival stratified by AMscore levels in patients treated by adjuvant CT/RCT (D), CT (E) or RT (F), respectively. CT: chemotherapy; CRT: chemoradiotherapy. [file Image_1.tif]

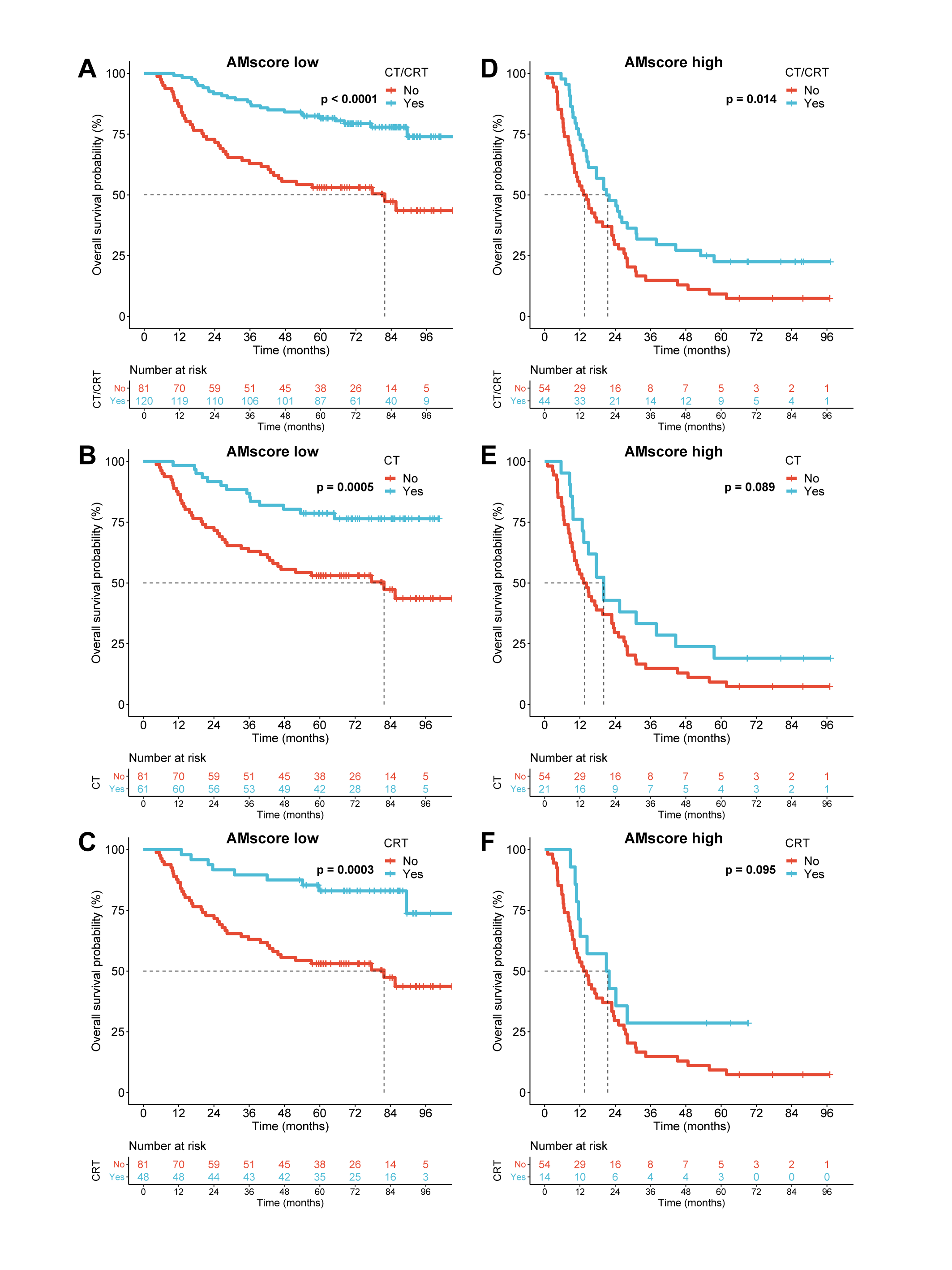

Supplement: Supplementary Figure 2 — The prognostic or predictive AMscore and the overall survival benefit of adjuvant chemotherapy or chemoradiotherapy in the ACRG cohort. (A–C): Overall survival (OS) benefit was significant in the AMscore low subgroup for CT/CRT (A), CT (B), and CRT (C), respectively. (D–F): Compared with the results in the AMscore low subgroup, OS benefit was substantially decreased in the AMscore high subgroup for CT/CRT (D), CT (E), and CRT (F), respectively. ACRG: Asian Cancer Research Group; CT: chemotherapy; CRT: chemoradiotherapy. [file Image_2.tif]

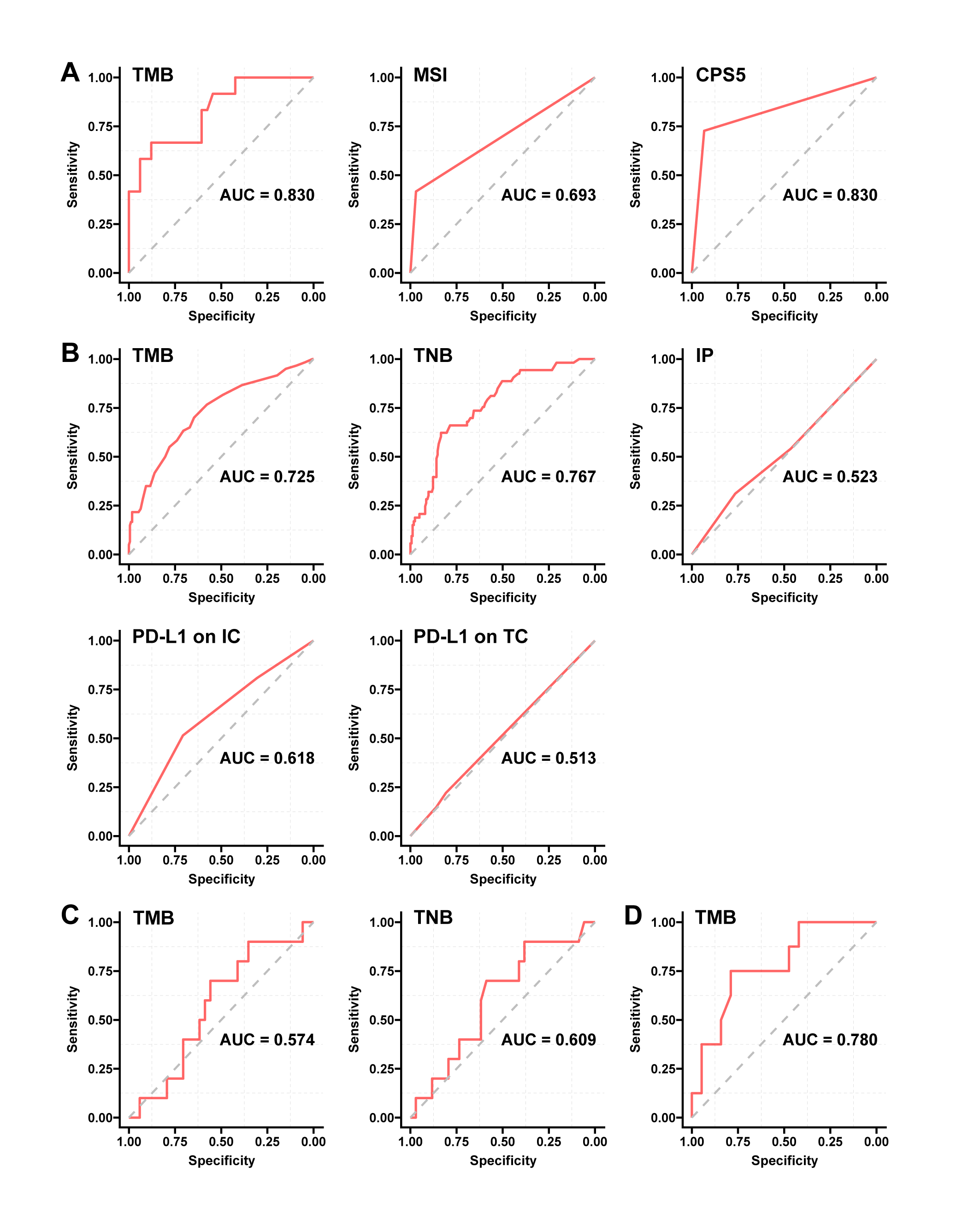

Supplement: Supplementary Figure 3 — Immunotherapy response prediction by classic biomarkers. (A–D): The receiver operating characteristic curve for response prediction by classic biomarkers in the immunotherapeutic gastric cancer (A), urothelial cancer (B), melanoma (C), and non-small-cell lung cancer (D) cohorts, respectively. TMB: tumor mutation burden; TNB: tumor neoantigen burden; MSI: microsatellite instability; CPS: combined positive score; IP: immune phenotype; IC: immune cells; TC: tumor cells. [file Image_3.tif]

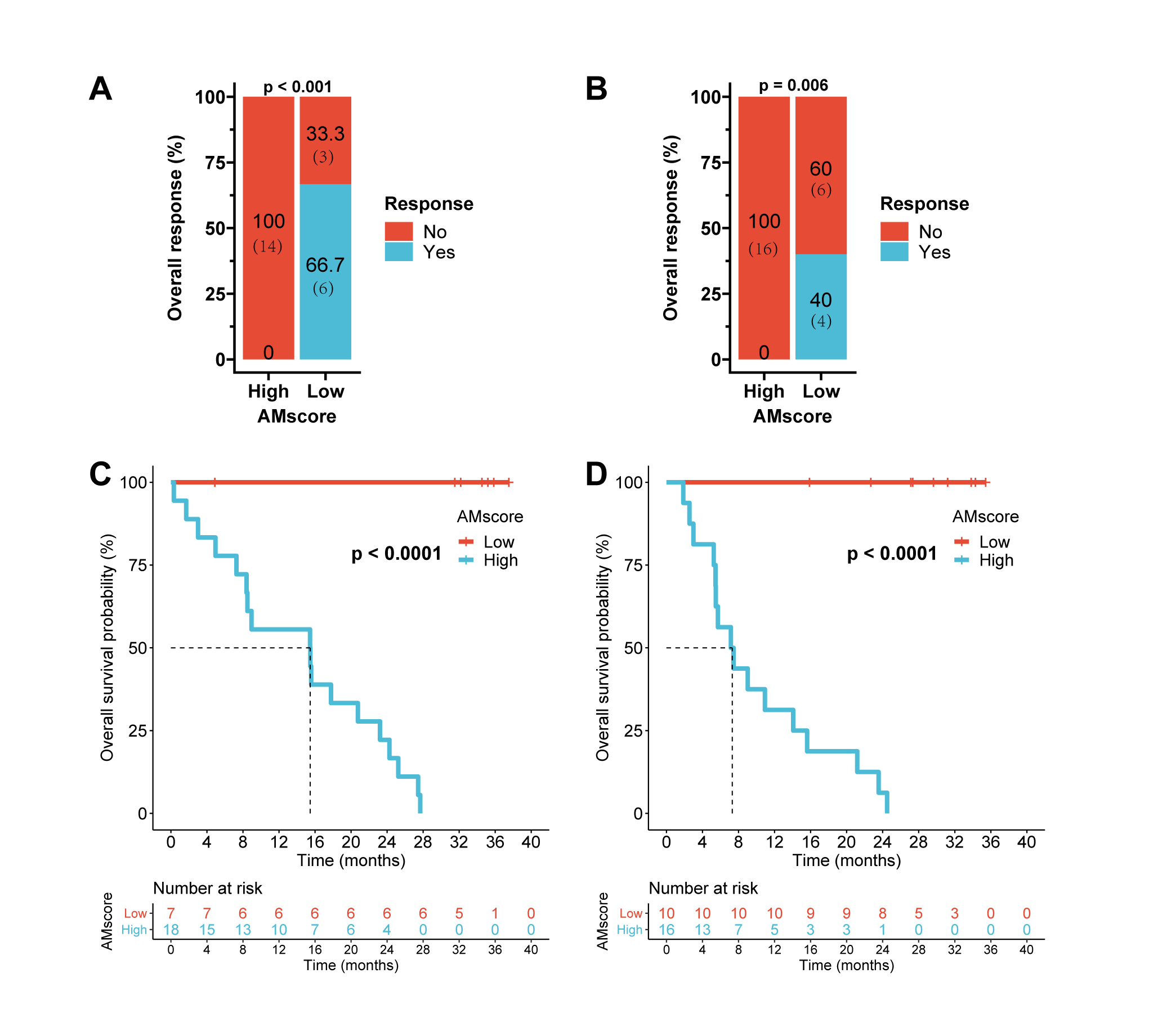

Supplement: Supplementary Figure 4 — Subgroup analysis in the melanoma cohort. (A, B): Overall response rates according to AMscore in the first-line (A) and second-line immunotherapy (B). (C, D): Overall survival according to AMscore in the first-line (C) and second-line immunotherapy (D). [file Image_4.tif]

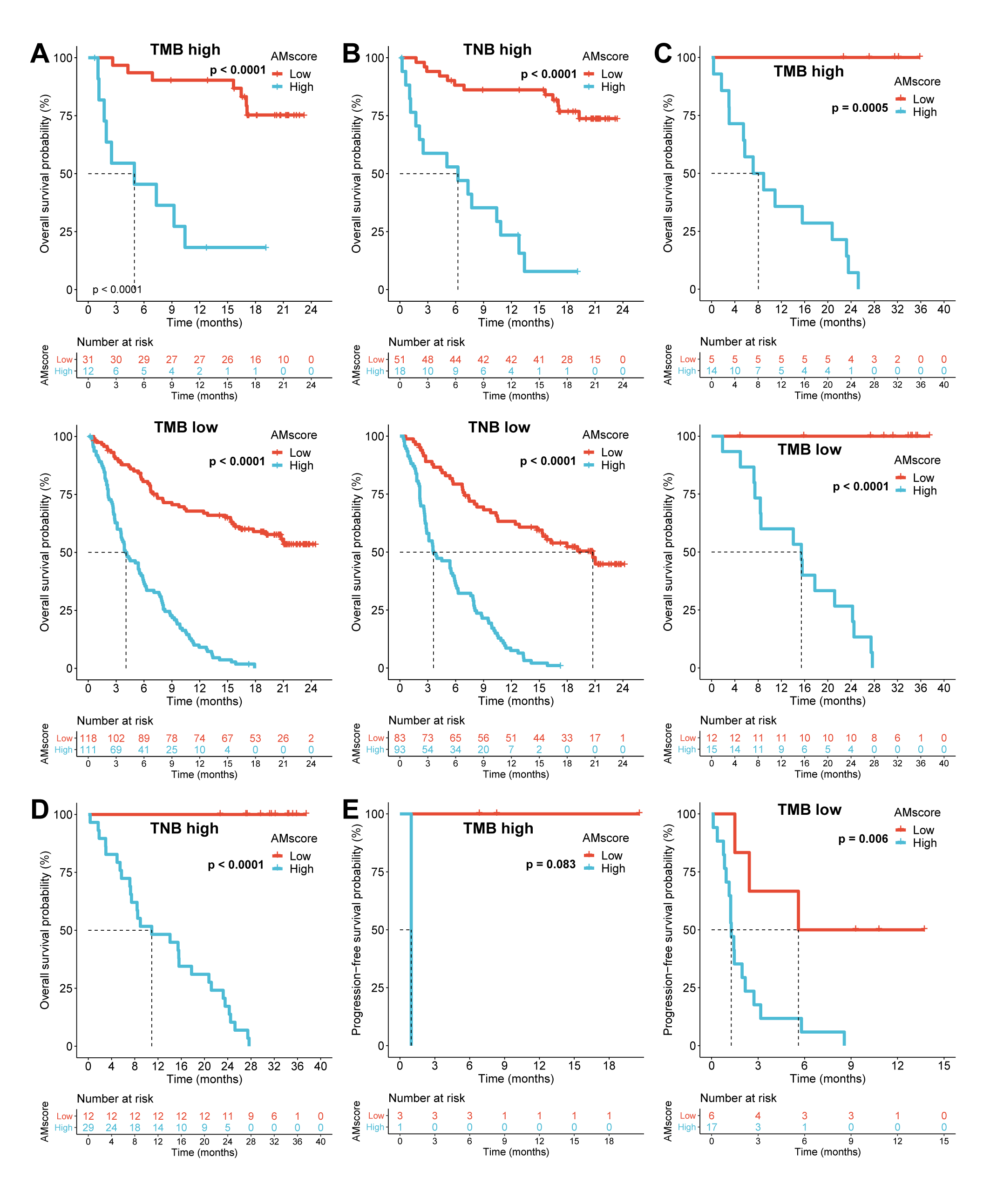

Supplement: Supplementary Figure 5 — Overall survival of immunotherapy stratified by the prognostic or predictive AMscore according to TMB or TNB levels. (A, B): the gastric cancer cohort; (C–E): the urothelial cancer (C), melanoma (D; all TNB low samples were also AMscore low), and non-small-cell lung cancer (E) cohorts, respectively. TMB: tumor mutation burden; TNB: tumor neoantigen burden. [file Image_5.tif]

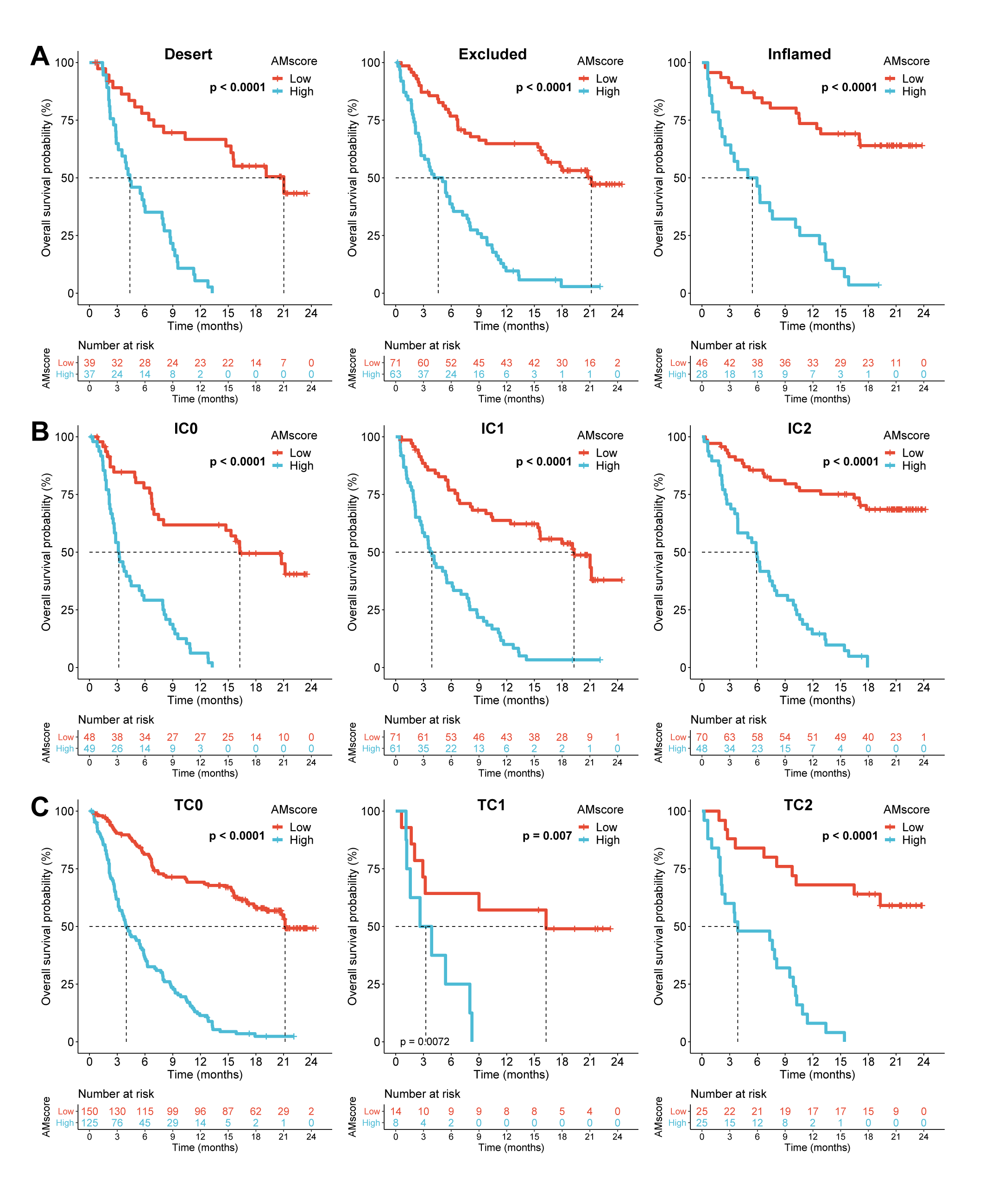

Supplement: Supplementary Figure 6 — Overall survival of immunotherapy stratified by the prognostic or predictive AMscore according to immune phenotype and PD-L1 expression in urothelial cancer. (A): immune phenotype. (B): PD-L1 expression on immune cells (IC). (C): PD-L1 expression on tumor cells (TC). [file Image_6.tif]

T16090527635

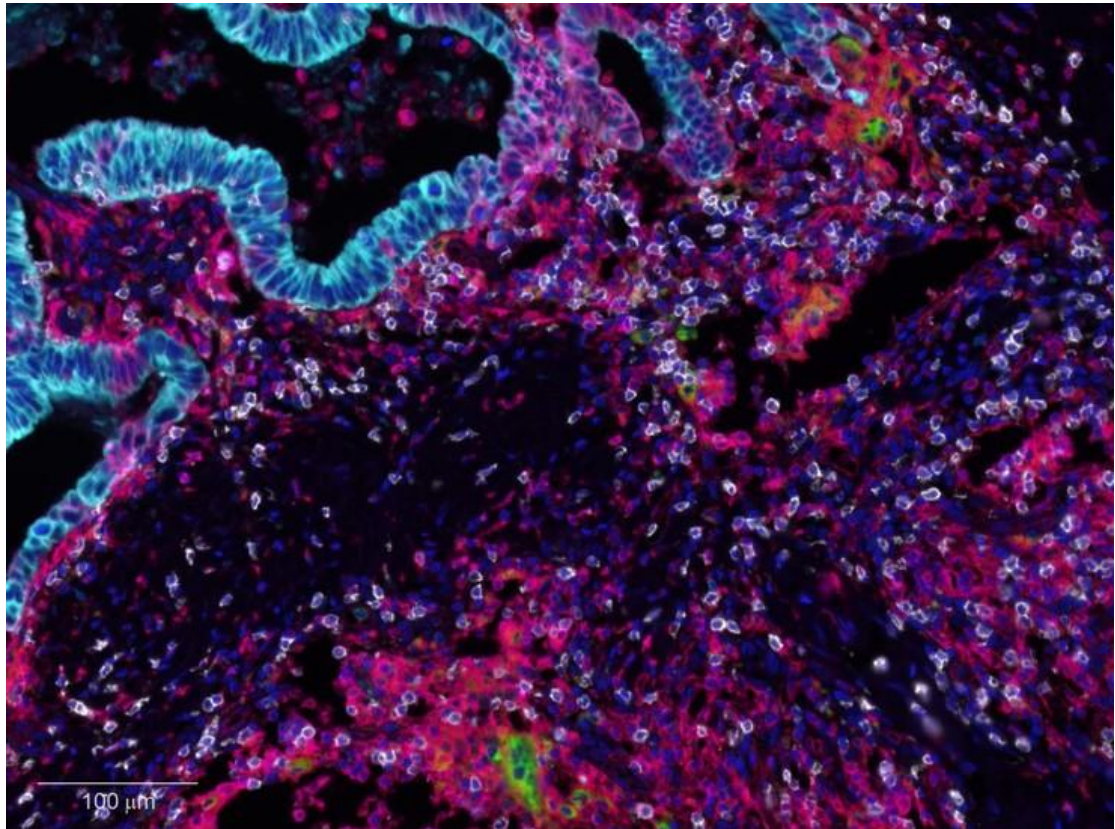

T16090527587

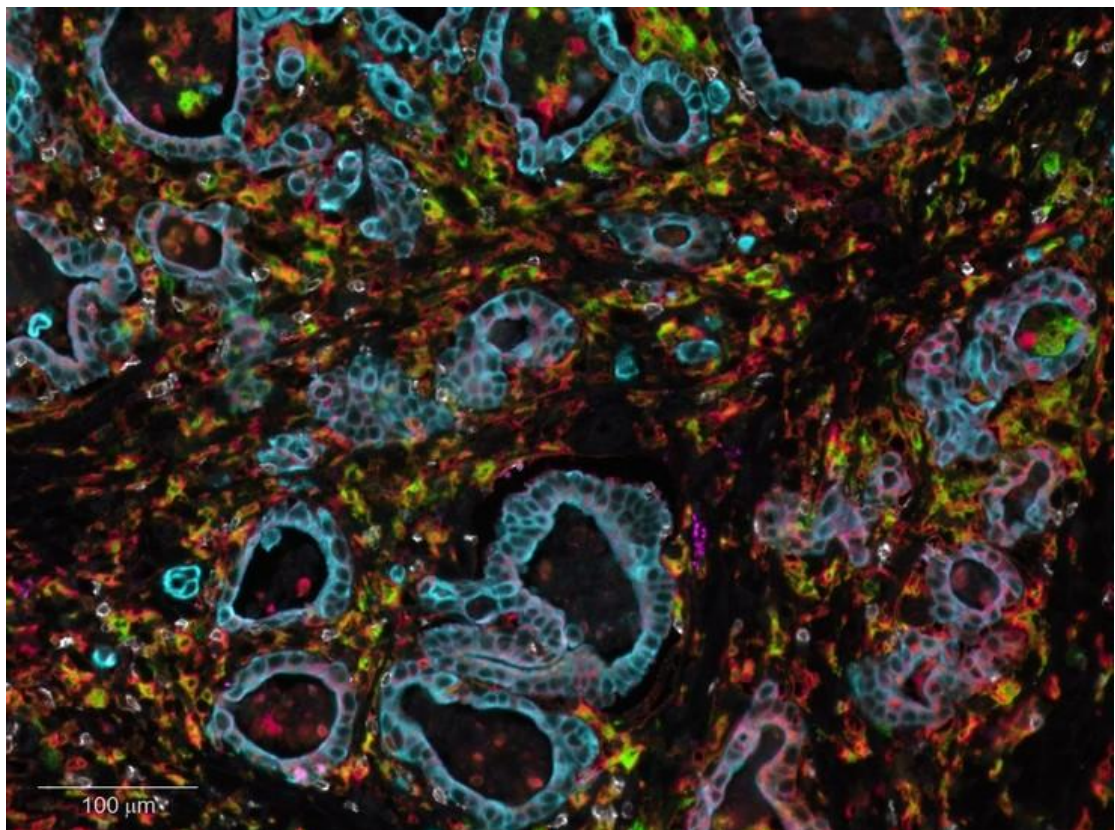

T16090527623

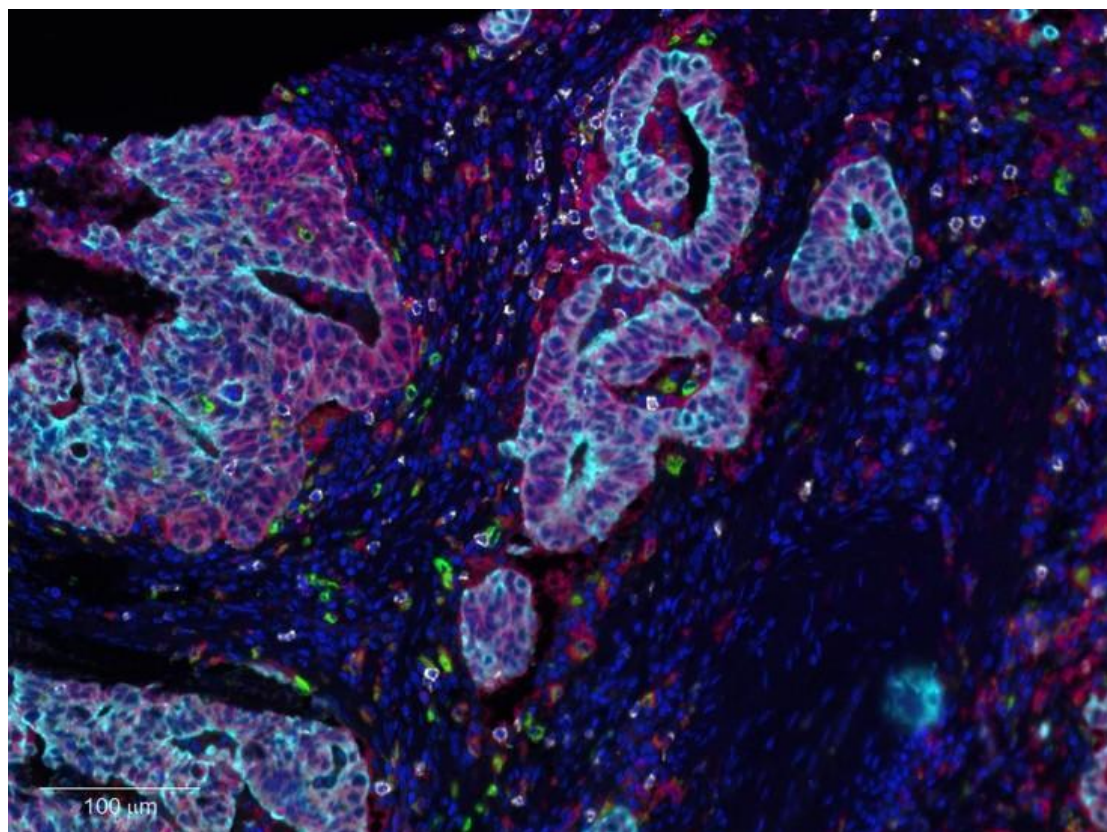

T16090527633

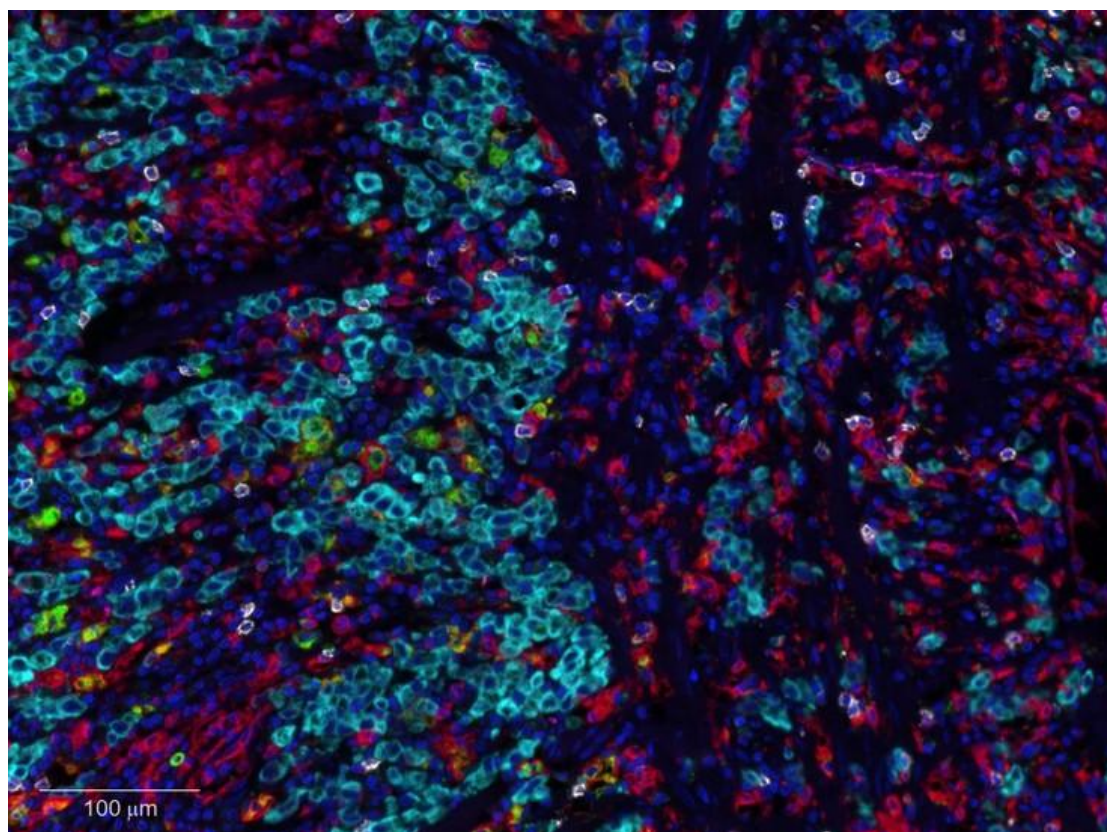

T16090527619

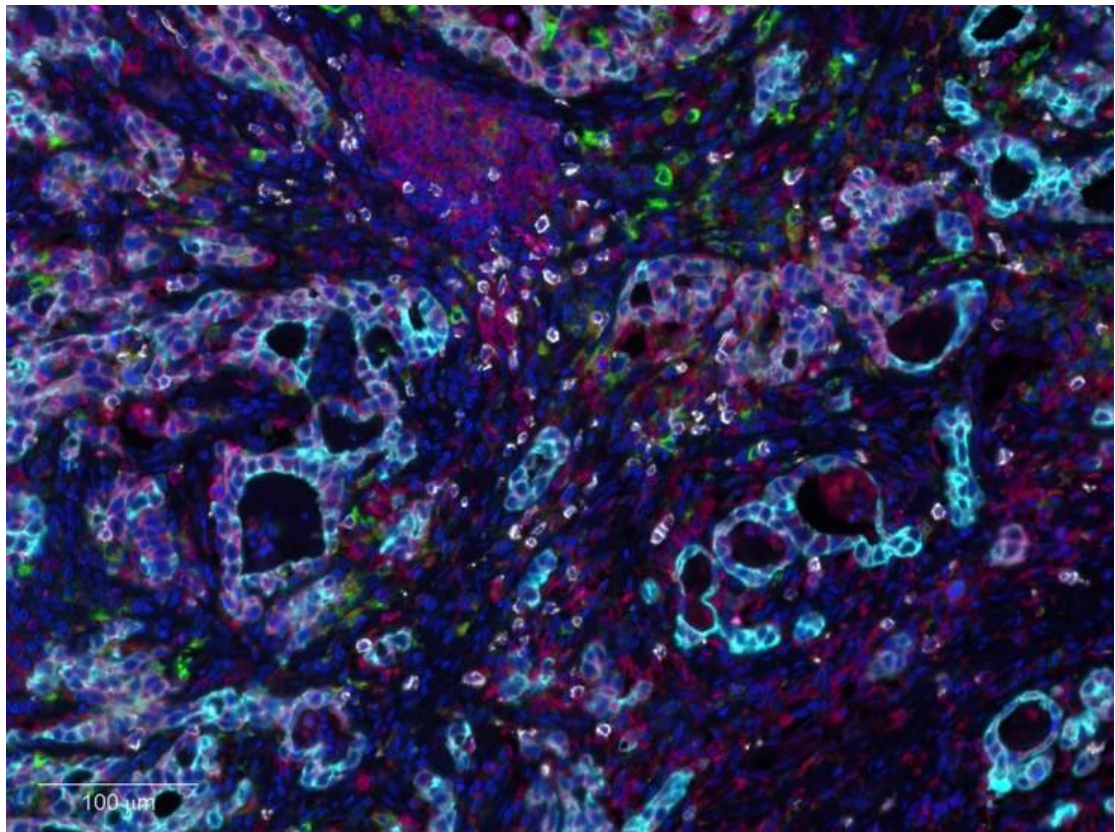

T16090527637

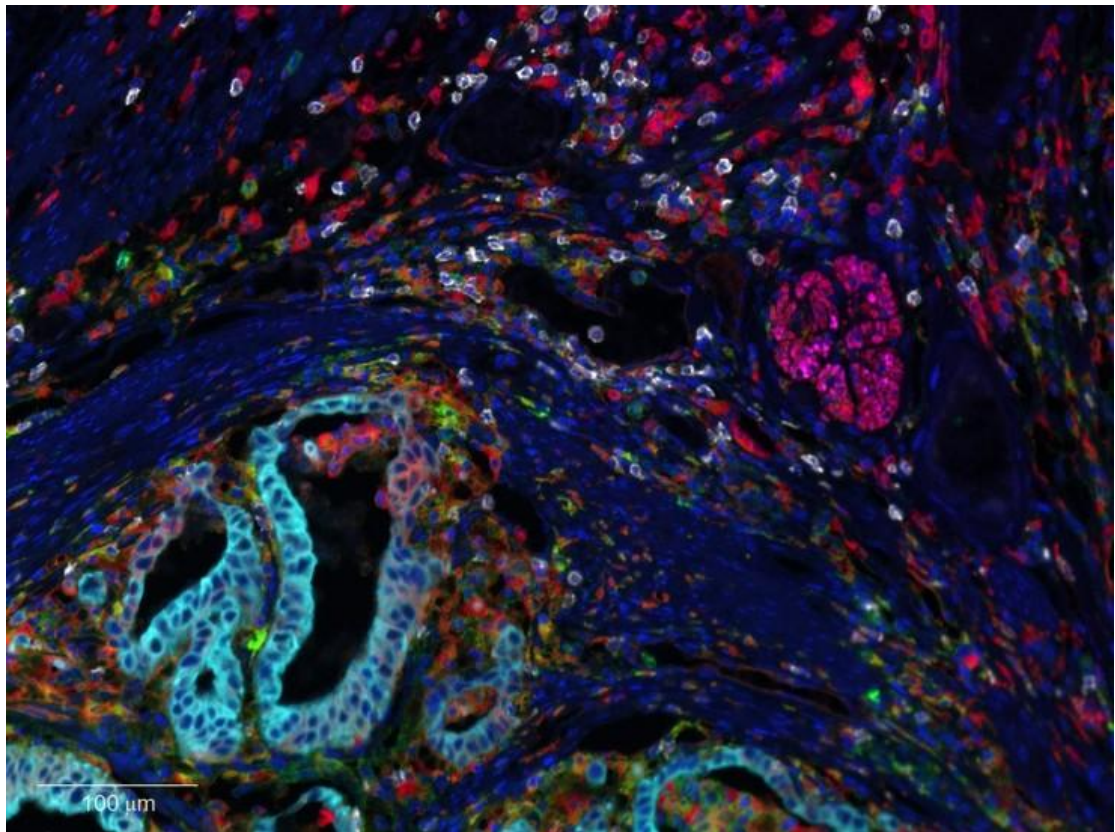

T16090527597

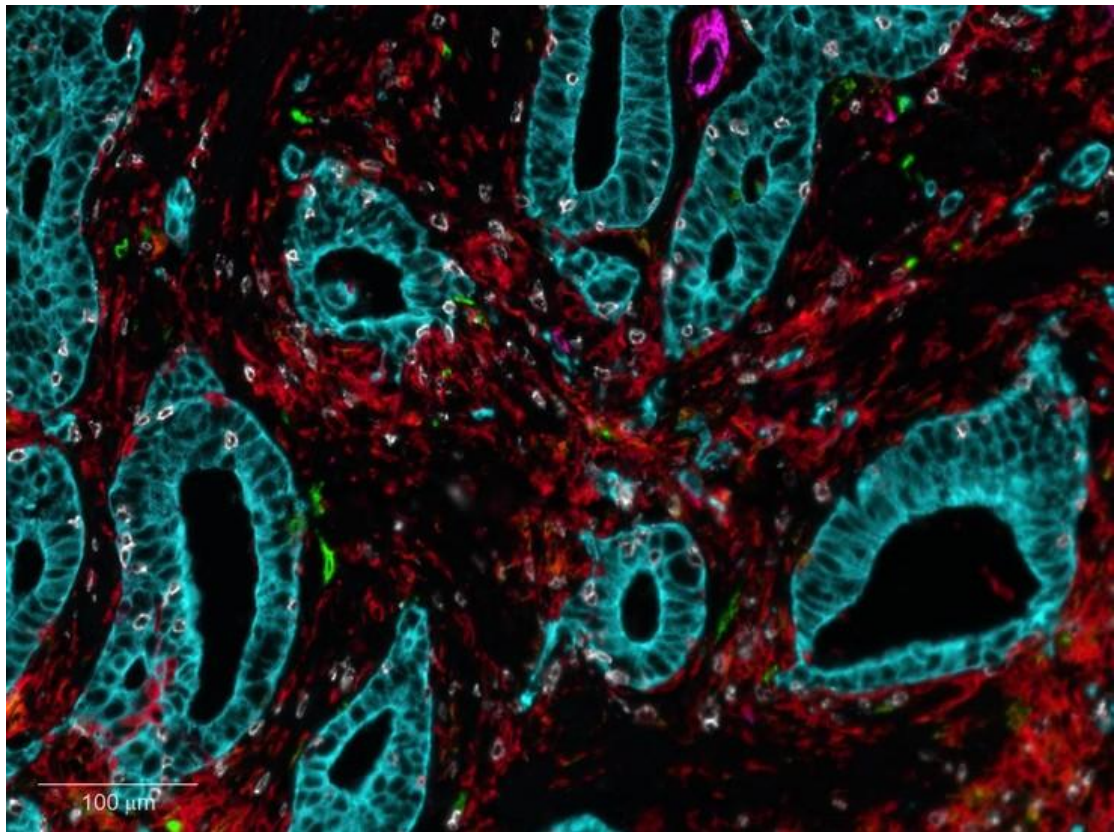

T16090527599

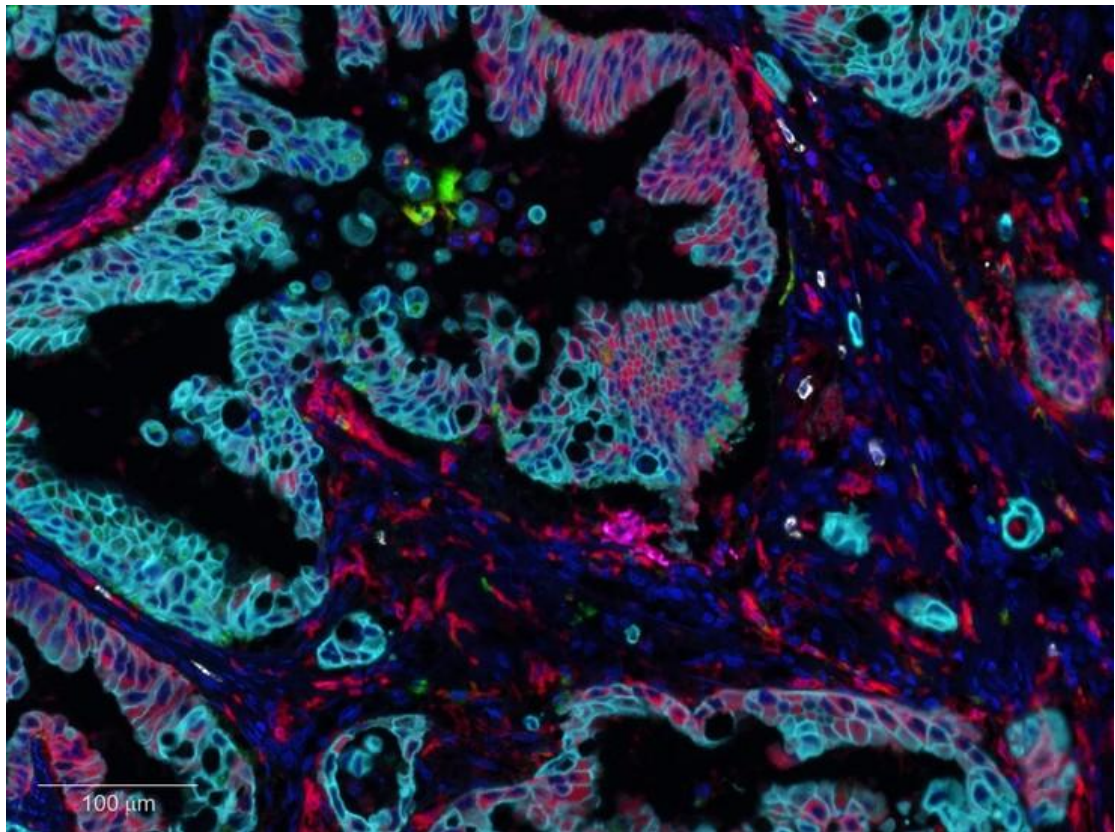

Supplement: Supplementary file 7 [file DataSheet_1.pdf]
