# Supplementary material for: Association of genes in hereditary metabolic diseases with diagnosis, prognosis, and treatment outcomes in gastric cancer
Source: Front Immunol. 2023 Nov 9;14:1289700. doi: 10.3389/fimmu.2023.1289700 (PMC10665511; doi:10.3389/fimmu.2023.1289700)
Supplement: Supplementary file 8 [file DataSheet_2.docx]

**Fig 1A and 1B**

library("Boruta")

data<-read.table("Fig1A.csv", head=T,sep = ",",stringsAsFactors = F,row.names =1)

set.seed(1)

Boruta.Ozone <- Boruta(data$tissue_type ~ ., data = data, doTrace = 0, ntree = 500)

plot(Boruta.Ozone)

getConfirmedFormula(Boruta.Ozone)

**Fig1C and 1D**

library(ggpubr)

library(ggplot2)

library("ggsci")

library(ggprism)

data<-read.table("Fig1C.csv", head=T,sep = ",",stringsAsFactors = F,row.names =1)

ggboxplot(data, x="gene", y="exp", color = "tissue", palette = "npg", add = "jitter")+

stat_compare_means(aes(group=tissue), method = "t.test",paired = TRUE, label = "p.format",size=4,fontface = "bold")+

theme(axis.text.x=element_text(size=16,angle=45,hjust=1,colour="black"),

axis.title.x=element_text(size=16,colour="black"),

axis.text.y=element_text(size=16,colour="black"),

axis.title.y=element_text(size=16,colour="black"),

legend.text= element_text(size=16, colour="black"),

legend.title=element_text(size=16,colour="black"))+

xlab("Gene")+ylab("mRNA expression")

**Fig 1E**

library(ggplot2)

library("ggsci")

library(ggprism)

data<-read.table("Fig1E.csv", head=T,sep = ",",stringsAsFactors = F,row.names =1)

ggpaired(data, x="tissue", y="AMscore", fill="tissue",id = "paired",

add="jitter",line.color = "gray", line.size = 0.5,

palette="npg",

xlab=" ",

ylab="Diagnostic score",

legend.title=" ",show.legend = F) +

theme_prism()+

theme(legend.position = 'none')+

stat_compare_means(method = "t.test",paired = TRUE, comparisons=list(c("T", "N")))

**Fig 1F and 1G**

library(pROC)

library(ggplot2)

library("ggsci")

library(ggprism)

data<-read.table("Fig1F.csv", head=T,sep = ",",stringsAsFactors = F,row.names =1)

roc.list <- roc(tissue ~ AMscore, data = data)

ggroc(roc.list, size = 1.2,colour = "red",alpha=.6)+

theme(axis.text.x=element_text(size=16,colour="black"),

axis.title.x=element_text(size=16,colour="black"),

axis.text.y=element_text(size=16,colour="black"),

axis.title.y=element_text(size=16,colour="black"),

legend.text= element_text(size=16, colour="black"),

legend.title=element_text(size=16,colour="black"))+

ggsci::scale_color_npg()+theme_prism()+

geom_segment(aes(x = 1, xend = 0, y = 0, yend = 1), color="grey", linetype="dashed",size=1)+

theme(panel.grid.minor = element_line(colour = "grey", linetype="dashed",size = 0.1),

panel.grid.major = element_blank(),

panel.background =element_blank(),axis.line = element_line(colour = "black"))+

annotate("text", x=0.25, y=0.4, label="AUC = 0.991",size=6, fontface="bold")

**Fig 2A**

library(survminer)

library(survival)

library(ggplot2)

library(ggstatsplot)

library(viridis)

Coxoutput <- read.csv("Fig2A.csv")

ggplot(data=Coxoutput,aes(x=HR,y=gene,color=pvalue))+

geom_errorbarh(aes(xmax=upper,xmin=lower,color = pvalue),height=0,size=1.3)+

geom_point(aes(x=HR,y=gene),size=7,shape=18)+

geom_vline(xintercept = 1,linetype='dashed',size=1.2)+

scale_x_continuous(breaks = c(0.3,1,2))+

coord_trans(x='log2')+

ylab("Gene")+

xlab("Hazard ratio")+

labs(color="P value",title ="" )+

scale_color_viridis(option="plasma")+

theme(panel.grid =element_blank())+

theme_bw(base_size = 12)+

theme(panel.grid =element_blank(),

axis.text.x = element_text(face="bold", color="black", size=16), axis.text.y = element_text(face="bold", color="black", size=16),

axis.title.x = element_text(face="bold", color="black", size=16),

axis.title.y = element_text(face="bold",color="black", size=16),

legend.text= element_text(face="bold", color="black", size=14),

legend.title = element_text(face="bold", color="black", size=14),

panel.border = element_rect(colour = 'black',size=1.4))

**Fig 2B**

An R packet is used, contact details: [figureya@126.com](mailto:figureya@126.com)

**Fig 2C-2E**

library(survminer)

library(survival)

data<-read.table("Fig2C.csv",head=T,sep = ",",stringsAsFactors = F)

fit<-survfit(Surv(os,death)~ AMscore, data = data)

ggsurvplot(fit,data = data,

conf.int = F,

break.time.by = 12,

risk.table.col = "strata",

pval = T,

surv.median.line = "hv",

fun = "pct",

risk.table = TRUE,risk.table.y.text.col = TRUE,

size = 2,

censor.size = 6,

palette = c("npg"),

xlab = "Time (months)",

ylab = "Overall survival probability (%)",

ylim = c(0,100),

font.legend = c(16),

fontsize = 5, risk.table.height = 0.20,

font.x = c(16, "black"),

font.y = c(16, "black"),

font.tickslab = c(16, "black"),

legend = c(0.15,0.15),

legend.title = "AMscore",

legend.labs = c("Low","High"))

**Fig 3A-3C**

**Similar to Fig 2C-2E**

**Fig 3D-3H**

library("forestplot")

data <- read.csv("Fig3D.csv", stringsAsFactors=FALSE)

tabletext <- cbind(c("\nCharacteristic",data$Variable),

c("Hazard Ratio\n(95% CI)",ifelse(!is.na(data$P.value), paste(format(data$Point.Estimate,nsmall=2)," (",format(data$Low,nsmall = 2)," to ",format(data$High,nsmall = 2),")",sep=""))),

c("\nP.value",data$P.value))

forestplot(labeltext=tabletext,mean=c(NA,data$Point.Estimate),

lower=c(NA,data$Low),upper=c(NA,data$High),

graph.pos=4,#图在表中的列位置

#graphwidth = unit(.4,"npc"),#图在表中的宽度比例

fn.ci_norm="fpDrawDiamondCI",#box类型选择钻石

col=fpColors(box="steelblue", lines="black", zero = "black"),#box颜色

boxsize=0.25,#box大小根据样本量设置

lwd.ci=2,ci.vertices.height = 0.1,ci.vertices=TRUE,#置信区间用线宽、高、型

zero=1,#zero线横坐标

lwd.zero=2,#zero线宽

grid = structure(c(data[1,]$Point.Estimate), gp = gpar(col = "black", lty=2,lwd=2)),#虚线(可多条)及其横坐标、颜色、线宽

#xticks = c(0.5, 1,1.5, 2),#横坐标刻度根据需要可随意设置

lwd.xaxis=2,#X轴线宽

xlab="Hazard Ratio",#X轴标题

hrzl_lines=list("2" = gpar(lwd=2, col="black"),#第三行顶部加黑线，引号内数字标记行位置

#"4" = gpar(lwd=60,lineend="butt", columns=c(1:4), col="#99999922"),#加阴影，弱项不建议使用

"14" = gpar(lwd=2, col="black")),#最后一行底部加黑线,""中数字为nrow(data)+5

txt_gp=fpTxtGp(label=gpar(cex=1.25),#各种字体大小设置

ticks=gpar(cex=1.25),

xlab=gpar(cex = 1.25),

title=gpar(cex = 1.25)),

#is.summary = c(T,rep(F,27)),#首行字体类型设置

lineheight = unit(2,"cm"),#固定行高

align=c("l","l","l"),#每列文字的对齐方式，偶尔会用到

#cex=10,

colgap = unit(0.3,"cm"),#列间隙

#mar=unit(rep(1.25, times = 4), "cm"),#图形页边距

new_page = T#是否新页

)

**Fig 4A**

An R packet is used, contact details: [figureya@126.com](mailto:figureya@126.com)

**Fig 4B**

library(pheatmap)

col <- colorRampPalette(c("#4DBBD5B2", "white", "#DC0000B2"))(256)

data<-read.csv("Fig4B.csv",head=T,sep = ",",stringsAsFactors = F,row.names =1)

df=as.matrix(data)

anno<-read.csv("anno1.csv",header = T,sep = ",",stringsAsFactors =F,row.names =1)

df<-df[,rownames(anno)]

pheatmap(mat=df,legend_side = "bottom",col=col,clustering_distance_rows = "correlation",annotation = anno, scale = "row",fontsize = 14, show_colnames = F,cluster_cols= F)

**Fig 4C**

library(ggplot2)

library("ggsci")

library(ggprism)

data<-read.csv("Fig4C.csv",header = T)

data<-data[order(data$EnrichmentScore,decreasing = F),]

data$order<-seq(1:nrow(data))

ggplot(data=data,aes(x=reorder(Name,order),y=EnrichmentScore,fill=Padj))+

geom_bar(stat='identity',position=position_dodge()) + coord_flip()+

scale_fill_gradient(low = 'red', high = 'blue')+

theme(axis.text.x=element_text(size=14,colour="black"),

axis.title.x=element_text(size=14,colour="black"),

axis.text.y=element_text(size=14,colour="black"),

axis.title.y=element_text(size=14,colour="black"),

legend.text= element_text(size=14, colour="black"),

legend.title=element_text(size=14,colour="black"))+

theme(strip.text.x = element_text(size=16,colour="black"))+

ylab("EnrichmentScore")+xlab("")

**Fig 5A-5F**

**Similar to Fig 2C-2E**

**Fig 6A, 6B, 6D, 6F and 6G**

library(ggplot2)

library("ggsci")

library(ggprism)

library("gridExtra")

data<-read.table("Fig6A.csv",head=T,sep = ",",stringsAsFactors = F)

p1=ggplot(data, aes(x = log(tmb), y = AMscore, colour = cohort))+

geom_point(alpha = .4, size = 8)+

theme(legend.key.size=unit(0.8,'cm'))+

guides(colour = guide_legend(override.aes = list(size=8)))+

theme(axis.text.x=element_text(size=14,colour="black"),

axis.title.x=element_text(size=16,colour="black"),

axis.text.y=element_text(size=14,colour="black"),

axis.title.y=element_text(size=16,colour="black"),

legend.text= element_text(size=14, colour="black"),

legend.title=element_text(size=14,colour="black"))+

stat_cor(data=data, method = "pearson", size=5)+

geom_smooth(se = T, method = lm, size = 1.5, formula = y ~ x,colour="yellow")+

scale_size_area()+theme_prism()+

scale_y_continuous(guide = guide_prism_minor())+

ylab("AMscore")+xlab("Log(TMB)")+

facet_wrap(~ cohort, scales="free", ncol=4)+

theme(strip.text.x = element_text(size=16,colour="black"))+

theme(strip.text.x = element_text(size=16,colour="black"))+

theme(panel.grid.major = element_blank(), panel.grid.minor = element_blank(),

panel.background =element_blank(),axis.line = element_line(colour = "black"))+

theme(legend.position = "none")

p1_npg = p1 + scale_color_npg()

grid.arrange(p1_npg)

**Fig 6C and 6E**

library(ggplot2)

library("ggsci")

library(ggprism)

data<-read.table("Fig6C.csv",head=T,sep = ",",stringsAsFactors = F)

df_p_val<-data%>%

rstatix::group_by(gene)%>%

rstatix::wilcox_test(AMscore~MSI)%>%

rstatix::add_xy_position()

ggplot(data,aes(x=MSI,y=AMscore))+

geom_boxplot(aes(fill = MSI))+

scale_fill_npg()+

ylab("Microsatellite status")+xlab("AMscore")+

theme_prism()+

theme(legend.position = "none")+

facet_wrap(~ cohort,scales="free",ncol=4)+

theme(strip.text.x = element_text(size=16,colour="black"))+

theme(axis.text.x=element_text(size=16, colour="black"),

axis.title.x=element_text(size=16, colour="black"),

axis.text.y=element_text(size=16, colour="black"),

axis.title.y=element_text(size=16,colour="black"),

legend.text= element_text(size=16, colour="black"),

legend.title=element_text(size=16, colour="black"))+

scale_y_continuous(guide = guide_prism_minor())+

add_pvalue(df_p_val,fontface = "bold",label.size = 5)

**Fig 7A**

Not related to R

**Fig 7B-7D**

**Similar to Fig 6A**

**Fig 8A-8D**

**Similar to Fig 1F**

**Fig 8E-8G**

**Similar to Fig 2C**
